# Magneto-Responsive Chiral Optical Materials: Flow-Induced Twisting of Cellulose Nanocrystals in Patterned Magnetic Fields

**DOI:** 10.1021/acsnano.4c05320

**Published:** 2024-09-05

**Authors:** Minkyu Kim, Jisoo Jeon, Kellina Pierce, Daria Bukharina, Woosung Choi, Jinyoung Choi, Dhriti Nepal, Michael E. McConney, Timothy J. Bunning, Vladimir V. Tsukruk

**Affiliations:** † School of Materials Science and Engineering, Georgia Institute of Technology, Atlanta, Georgia 30332, United States; ‡ Department of Chemical Engineering, Dankook University, Yongin 16890, Republic of Korea; § Air Force Research Laboratory, Wright-Patterson Air Force Base, Ohio 45433, United States

**Keywords:** tunable chiral organization, induced chirality inversions, magnetic cellulose nanocrystals, liquid crystalline phase, patterned magnetic field and vortices

## Abstract

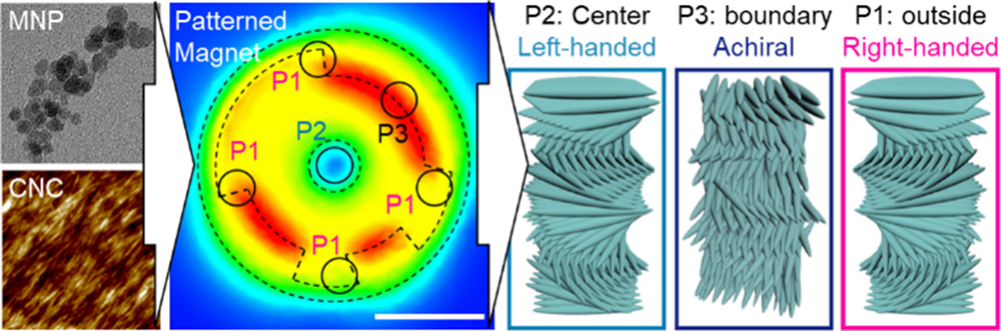

Magnetic fields have been used to uniformly align the lyotropic chiral nematic (cholesteric) liquid crystalline (LC) phase of biopolymers to a global orientation and optical appearance. Here, we demonstrate that, in contrast, weak and patterned magnetic field gradients can create a complex optical appearance with the variable spatial local organization of needle-like magnetically decorated cellulose nanocrystals. The formation of optically patterned thin films with left- and right-handed chiral and achiral regions is observed and related to local magnetic gradient-driven vortices during LC suspension flow. We trace the localized flow directions of the magnetically decorated nanocrystals during evaporation-induced assembly, demonstrating how competing evaporation and field-induced localized flow affect the twisted organization within magnetically induced vortices. The simulations suggested that localized twisting inversion originates from the interplay between the direction and strength of the local-depth-related magnetic gradients and the receding front through peripheral magnetic gaps. We propose that this finding will lead to magnetically patterned photonic films.

## Introduction

Control of global and local chirality is critically important for diverse applications such as enantioselective synthesis, chiro-optical photonics, chiral plasmonic appearance, or chiral electronic superconductance.^1−5^ Among the promising bioenabled chiroptical photonic material components, needle-like rigid cellulose nanocrystals (CNCs) with natural lyotropic chiral nematic organization are widely considered due to their self-assembled helicoidal ordering with selective light reflection and circular dichroism (CD).^6−8^

The chiral nematic phase of these CNC suspensions occurs at modest critical concentrations, between 3 and 7 wt %, and long-range helicoidal organizations form during evaporation-induced self-assembly (EISA).^9−11^ These films selectively reflect left-handed circular polarized light, thereby exhibiting rich colors by reflection and circular polarization activity.^12,13^ Numerous efforts have been made to control the chirality of self-assembled CNCs since it is at the core of the chiro-optical properties of CNC films.^14−16^ Among recent studies on tunable chiral CNC-based materials, MacLachlan and co-workers demonstrated that CNC-elastomer composites can shift from a left-handed to a pseudonematic alignment due to tensile strain.^17^ Layer-by-layer printing can induce preprogrammed helical organization.^18^ Ounaies and co-workers have demonstrated that the anisotropic order of the chiral nematic phase of CNCs can be tuned by applying a magnetic field (0.7 T) to both aqueous and nonaqueous suspensions.^19^ Additionally, Xu and co-workers reported that magnetic field-directed self-assembly of CNC/Fe_3_O_4_ results in left-handed chiral nematic CNCs formed with a homeotropic concentric texture in planar films.^20^

Since a modestly strong magnetic field (1–28 T) is required to control the assembly behavior of CNCs,^21,22^ magnetic nanoparticles (MNPs) are usually employed for decoration in these studies. However, chirality inversion in self-assembled CNCs (from natural left-handed to right-handed) has rarely been observed. As one of the few examples, concentrated cellulose acetate, ranging from 27 to 40 wt %, in trifluoroacetic acid initially exhibits a left-handed structure, which reverses to a right-handed structure when trifluoroacetate groups are grafted onto the CNC backbone.^23^ In our recent study, we demonstrated that a uniform uniaxial structure forms by slow drying of magnetic CNC suspensions placed above a permanent coin magnet.^14^ This behavior is due to magnet-induced radial shearing within circular flow with a high rate (10–20 μm/s). However, this high shearing resulted in a unialigned orientation with suppressed helical organization.

**Figure 1:**
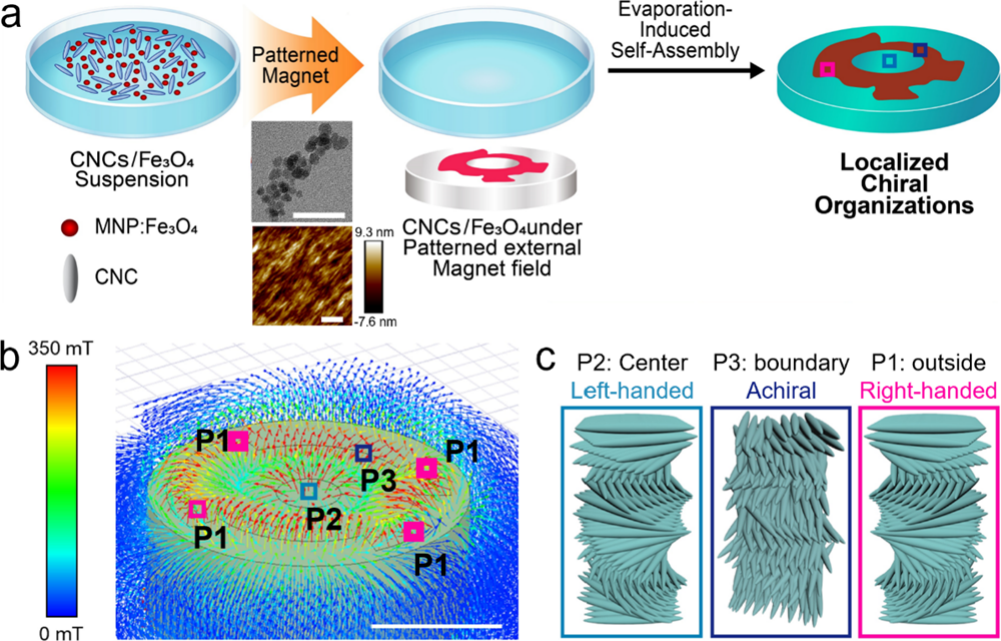
Schematic procedure for controlling localized chirality inversion of self-assembled CNCs using magnetic field gradient patterns. (a) Preparation of the CNC/MNP film by evaporating within a patterned magnetic field, and images of magnetic particles and CNCs. Inset images are TEM image of MNPs and AFM image of CNCs. Scale bars for TEM and AFM are 500 nm and 200 nm, respectively. (b) Perspective view of a simulated circular patterned magnetic field and (c) corresponding alignment of CNCs depending on the position of the magnetic field revealed in this study. Scale bar in (b) is 1 cm.

In this study, we demonstrate that patterned magnetic fields under 300 mT with narrow peripheral magnetic gaps with local magnetic gradients induce inversion of the twisted structure, from left to right, of magnetically decorated CNCs. This reorganization occurs during the formation of thin films in a slow evaporation regime of chiral nematic suspensions in the presence of a circular patterned magnetic field with localized evaporation-induced vortex flows (Figure 1). We traced in real time the flow directions of the magnetically decorated CNCs during evaporation-induced assembly to show evaporation-/field-induced localized vortices. Localized right-handed chirality was observed independently with CD spectra and Mueller matrix analysis of spectroscopic ellipsometry data and confirmed by morphological observations and simulation of a magnetic field gradient within localized vortices. Overall, we suggest that localized magnetic vortices can control the local twisted organization with controlled handedness in magnetically decorated CNCs, triggering diverse local helicity appearances across large-area thin films.

## Results and Discussion

For the preparation of magnetic CNC films, Fe_3_O_4_ MNPs were mixed with CNCs aqueous solution and thoroughly sonicated (Figure 1a; see details in Methods) according to our recent study.^14^ The Fe_3_O_4_ MNPs (ferrites with a spinel structure^24,25^) were synthesized by a reaction between ferrous (Fe^2+^) and ferric (Fe^3+^) ions in a base solution, followed by mixing them with citric acid to obtain a surface with carboxyl groups (see Methods).^14,26^ The average diameter of the MNPs was 7.0 ± 2.3 nm as derived from TEM images and dimension histograms (Figure S1). A hydrodynamic diameter around 100 nm means that hydrogen bonding between CNC and MNP is enough to promote flow-induced alignment of CNC (Figure S2).

**Figure 2:**
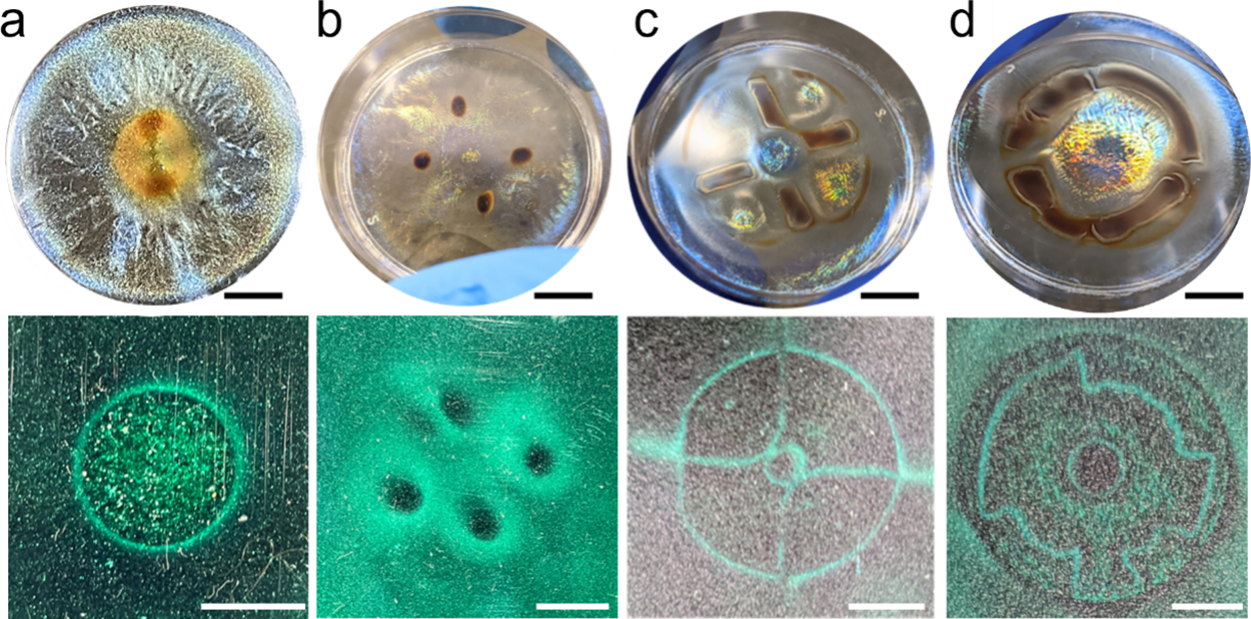
Variation of the pattern formed in CNC-MNP films (top) under different patterned magnetic fields (bottom). Photographs of diverse magnetic patterns in CNC/Fe_3_O_4_ composites (top panel) under different patterned magnetic fields (bottom panel): (a) coin, (b) four dots, (c) cross-shaped, and (d) circular-shaped magnetic patterns. Scale bars are 1 cm.

Patterned thin films were produced by drop-casting the mixed suspension into a Petri dish followed by slow EISA (Figure 1a). Suspension concentration and processing conditions were selected based on our previous studies to obtain thin (below 20 μm) uniform films across the large area.^14^ The suspension was placed directly on top of a circular permanent magnet that generates the patterned field shown in Figure 1b and then allowed to dry slowly (∼72 h). Initially, we explored different symmetrical and patterned magnets in comparison to coin magnets and evaporation with no magnets (Figures 2, S3, and S4; see more in the SI).

**Figure 3:**
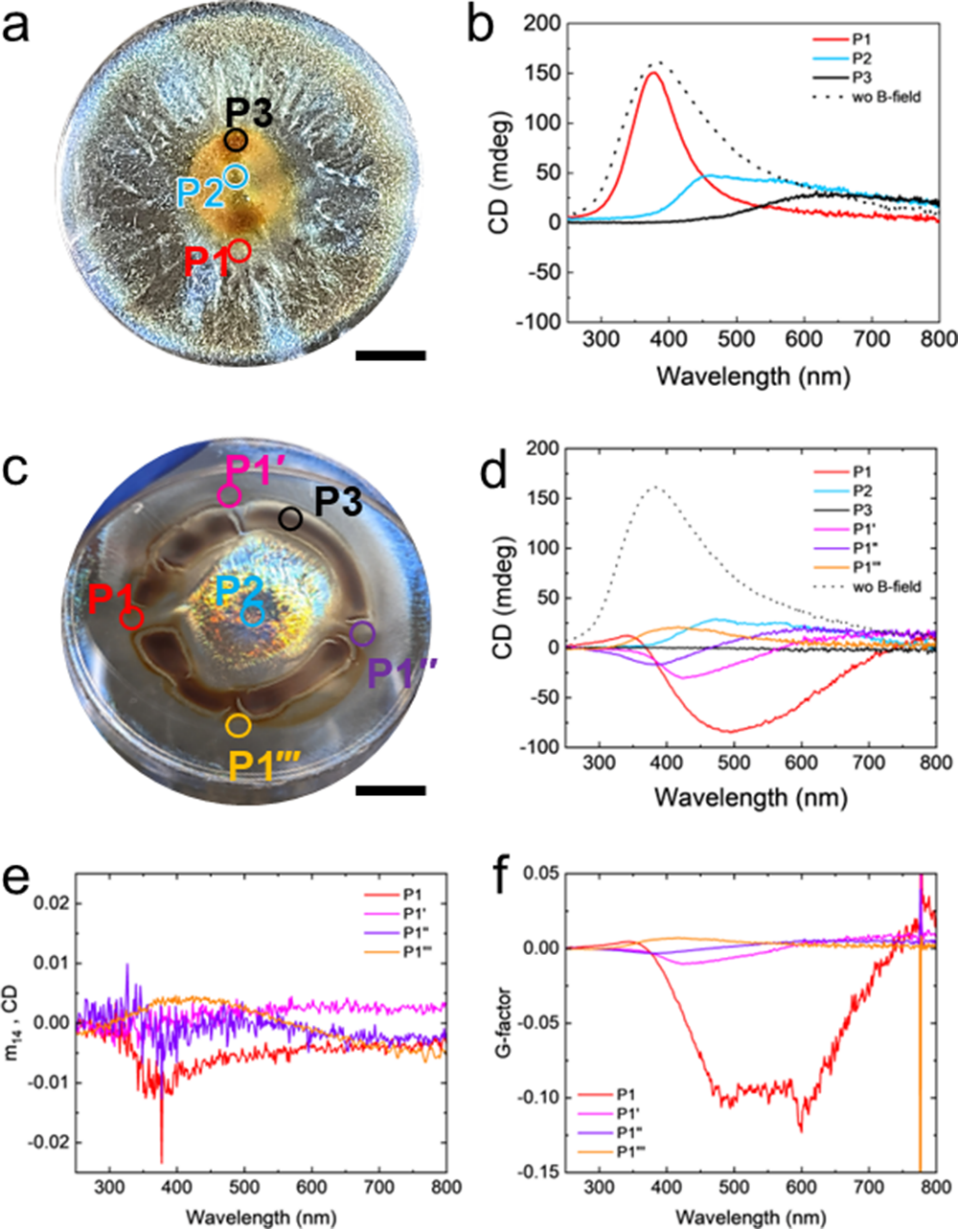
Chirality inversion of the CNCs through application of a patterned magnetic field. Photograph of CNC/MNP composite films evaporated on a (a) coin-shaped magnet and (c) a circular patterned magnet with selected three positions. P1, outside of the circular pattern; P2, center of the circular pattern; P3, boundary line of the magnetic pattern. Scale bars are 1 cm. CD spectras of the CNC/MNP film evaporated on a (b) coin-shaped magnet and (d) circular patterned magnet depending on the position of the films. The CD result of the composite film dried without a magnetic field is also plotted as a control in (b). (e) *m*_14_, CD component, measured from the Mueller matrix. (f) *G*-factor calculated at positions P1, P1′, P1″, and P1‴.

**Figure 4:**
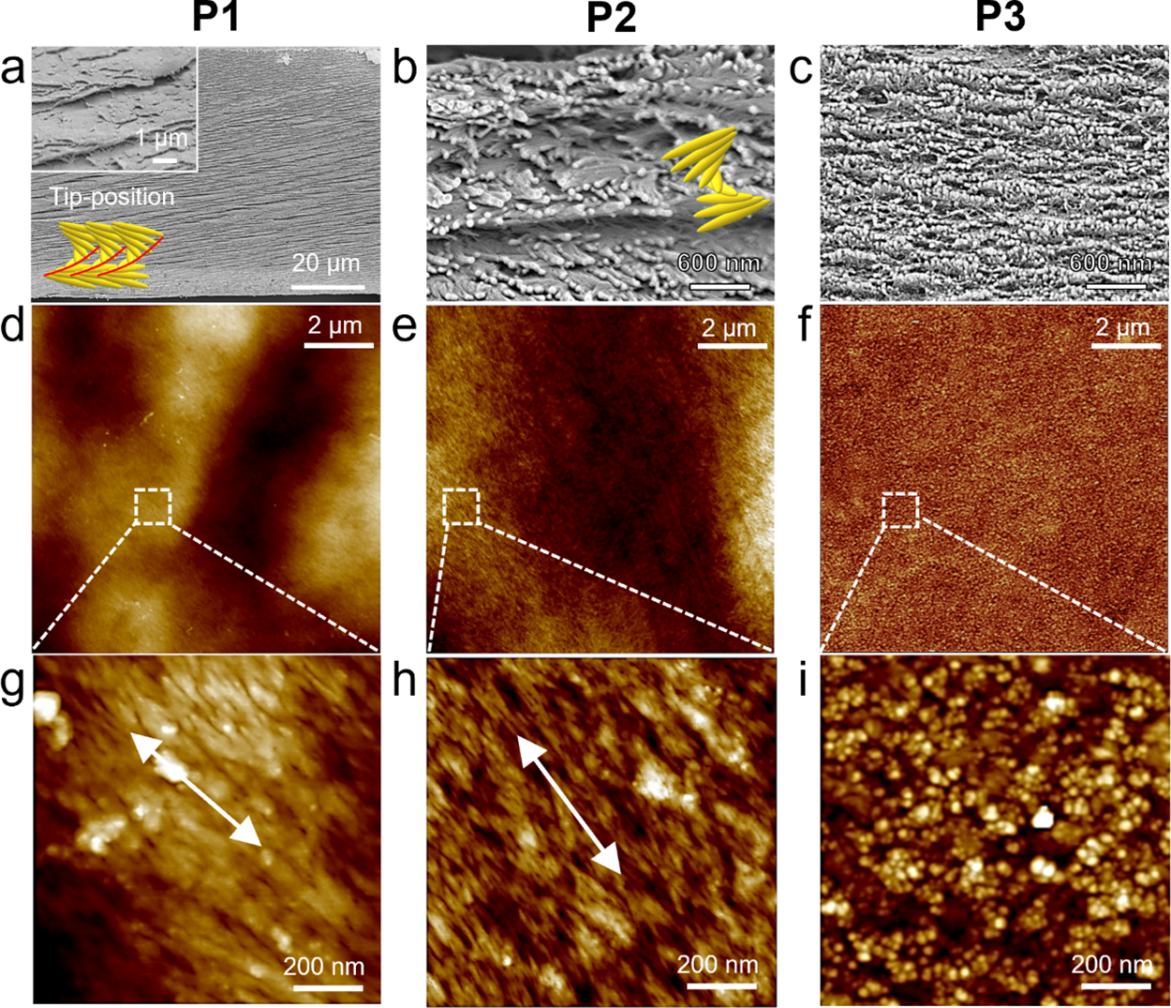
Morphology of the selected CNC surface areas depending on the position of the magnetic pattern. (a–c) SEM and (d–i) AFM images of the CNC/MNP film for selected three positions: P1–3. The arrows in (g) and (h) present the uniform orientation of the CNC on the film surface.

The pattern of each magnet was visualized by special magnetic films (see Methods) (Figure 2, bottom). The light-green color indicates that the magnetic field is directed toward horizontal directions, while the magnetic field is directed toward vertical directions in the dark-green-color area. Drying a CNC/MNP suspension under a complex magnetic field enables the patterning of the local iridescence, resulting in diverse patterned films with localized aggregation of MNPs where the magnetic flux density is higher at the periphery. For instance, a film evaporated on the asymmetrically patterned circular magnet displays asymmetric dark-brown ring patterns, while a film evaporated on the symmetrically patterned magnet features four dark-brown rectangles in a crossed arrangement. Optical properties and the local morphology of the patterned films are discussed below (Figures 3 and 4).

First, to evaluate the local chirality of films depending on the shape of the magnet and location of the magnetic field, CD was collected for different local regions for a coin magnet, a circularly patterned magnet with four different gaps, a four-dot magnet, a cross-patterned magnet, a ginkgo leaf-patterned magnet, and a wave-patterned magnet (Figures 3, S5, S6, and S7). However, the chiral inversion phenomenon was discovered only in narrow gaps of the circularly patterned magnet.

Indeed, for illustration, we analyzed the CNC/MNP film dried on the coin magnet with radial symmetry (Figure 3a). In this case, several local sites show only natural left-handed chirality (see discussions for other patterns in the SI). In the CNC/MNP film dried without a magnetic field, all locations show positive CD peaks with a red shift from 350 to 450 nm for the P2 position and to 640 nm for the P3 position (Figure 3a,b). The observed red shifts are associated with an increased pitch length due to intercalation of MNPs between CNC bundles as confirmed by independent UV–vis measurements (Figure S8).^14^

In striking contrast, the CNC/MNP composite films dried under a circular patterned magnetic field with narrow gaps exhibit diverse circular polarization properties. All, left-handed, right-handed, and nonchiral, organizations are observed at different spatial locations (see all designations in Figure 3c,d). Specifically, at the P3 location, where the concentration of aggregated MNPs is the highest, no chirality was observed (Figure 3d). At the P2 location in the vicinity of the magnet core, traditional left-handed chirality was observed within the iridescent area. However, nonconventional right-handed chirality was observed at the other, P1, P1′, and P1″ locations at different magnetic gaps.

As was reported, MNP-decorated CNCs possess a high refractive contrast that might produce negative CD signals due to strong light scattering.^27−30^ However, it is not the case here with a common positive CD signal observed for thin CNC/MNP films without a magnetic field (Figure 3b). To confirm the chiro-optical behavior at each location, we conducted multiple measurements of the CD using different CNC batches and obtained consistent results.

For patterned films, the CD spectra show similar trends, with negative peaks observed at P1, P1′, and P1″ positions and positive peaks at P1‴, indicating that the observed phenomenon is reproducible (Figure S9). Additionally, to verify the origin of the CD signal, a Mueller matrix analysis derived from spectroscopic ellipsometry was conducted at each position and CNC film (Figures 3e, S10, and S11). For polydomain samples, the depolarization index was close to 1 (Figure S12). Among the matrix elements, m_14_, which exhibits a CD component from the Mueller matrix analysis, follows closely the trend observed from the CD spectrometer, showing negative peaks at P1, P1′, and P1″ and positive peaks at P1‴ and uniform CNC films, thus confirming the origin of CD signals discussed above. The other matrix elements, circular birefringence (CB), negative linear dichroism (−LD), negative linear birefringence (−LB), negative linear dichroism′ (−LD′), and linear birefringence (LB′), are shown in Figure S11. Among them, diagonal elements (*m*_22_, *m*_33_) exhibit polarization states of light. The diagonal elements of each position reach 1, which means the polarization states will not be changed, while if they reach −1, the polarization states will be converted when light passes through the samples.^31^ Direct comparison of absolute values cannot be conducted due to the dimensionless unit of CD from spectroscopic ellipsometry, but comparison shows similar trends of the signals obtained by different experimental techniques (Figure S13). The other asymmetric patterns, ginkgo leaves, and wave-patterned magnets, which have gaps in their magnetic field, have no clear chirality inversion due to low flow rates during suspension evaporation across a large area without narrow gaps (Figure S7).

To evaluate the chirality strength of each position, we further calculated the g-factor as a measure of circular polarization asymmetry (Figure 3g).^31^ The calculated g-factor reaches its highest negative peak around −0.1 in the range 500–600 nm at the P1 location, comparable to CPL asymmetry in natural CNC films. Negative broad g-peak values with reduced intensity can still be observed at the P1′ and P1″ points (around −0.02), indicating a significant reduction in circular polarization asymmetry. Finally, the P1‴ location shows a traditional broad positive g-peak, indicating a return to traditional left-handedness. Further, the difference in chirality in film organization was confirmed by the observation of Bouligand morphologies of locally oriented CNC texture in SEM and AFM analyses as shown in Figures 4, S14, and S15.

Cross-sectional SEM images of films reveal that CNCs are assembled into right-handed, left-handed, and achiral structures of CNCs at P1, P2, and P3, respectively, thus confirming the transformation of helical organization suggested by CD peak inversion (Figure 4a–c). SEM image at the P1 location shows diagonal cracks toward the right upper direction in a single film, which is a common crack pattern of right-handed liquid crystal polymers (Figure 4a).^32,33^ There are no clear left-handed twisted structures in the enlarged SEM at the P1 location (Figure 4a, inlet). The red line in the schematic illustration of CNCs shows the CNC positions that induce upper-right diagonal cracks (Figure 4a). However, a clear left-handed Bouligand layered morphology is observed for the P2 position (Figure 4b). At P3, there are no noticeable patterns due to the random aggregation of MNPs (Figure 4c). The AFM images illustrate the highly oriented organization of MNP-decorated CNCs on the top surface of the film at P1 and P2 locations common for chiral nematic ordering (Figure 4d,e,g,h). At P3, only larger aggregated MNP structures were observed due to an excessive concentration of MNPs in the area of the high magnetic field (Figure 4f,i). In order to verify the composition of these areas, X-ray photoelectron spectroscopy (XPS) was also conducted in the P1, P2, and P3 areas (Figure S16). Among these locations, the P3 area showed the highest signals of Fe 2p, while the P2 area showed no Fe signal, indicating the P2 area has almost no MNPs aggregated at surfaces. The Fe 2p peaks at the P1 area were of intermediate height between those of P2 and P3, indicating the presence of some MNPs.

These XPS spectra indicate that MNPs are localized at the areas with a higher magnetic flux density, indicating the phase separation of MNPs in strong magnetic areas due to some MNPs that are loosely bonded with CNCs. The weak hydrogen bonding between MNP and CNCs may be attributed to possible Coulombic repulsion between the negatively charged carboxylic group on the MNP surface and the negatively charged sulfate group of CNC, which balances the hydrogen bonding between the carboxylic group of the MNP and the hydroxyl group of the CNC. As a control group, the CNC/MNP film, dried without a magnetic field, exhibits a uniform structural color similar to that of a pure CNC film (Figure S3). Furthermore, negligible patterns are observed when conventional CNC suspensions are dried on the patterned magnetic fields, as illustrated in Figure S4. Overall, all of these results show that CNC decoration with MNPs is essential to achieve their orientation in a weak magnetic field.

**Figure 5:**
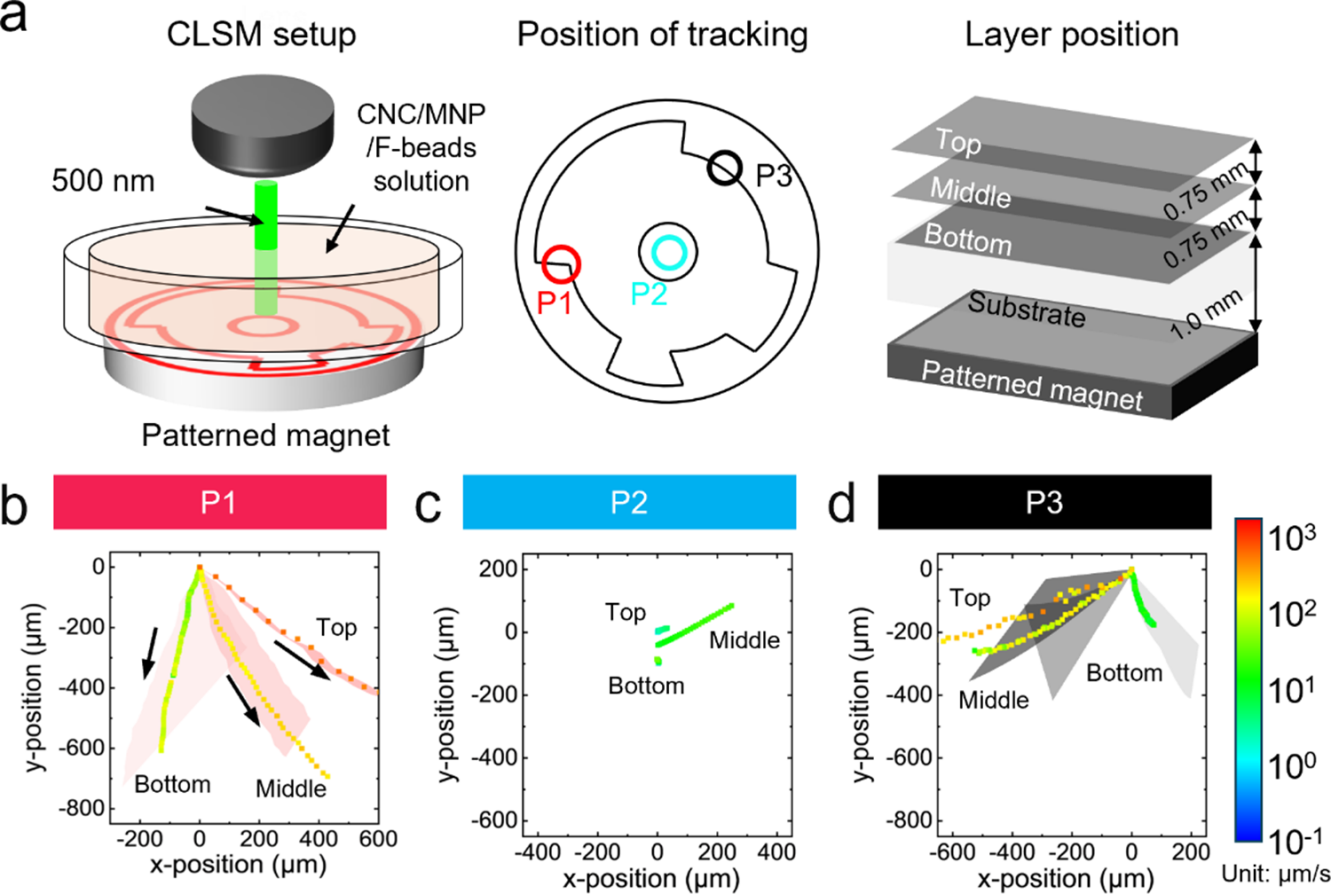
Flow directions and rates for magnetic gradient change depending on the position. (a) Schematic for the CLSM setup for tracking the flow direction of fluorescent microbeads in the CNC/MNP suspension at the bottom, middle, and top layers, respectively, under a circular patterned magnetic field. (b–d) Flow directions and flow rates at different elevations at three different positions, (b) P1, (c) P2, and (d) P3, that show a low flow rate in the middle (P2) and a very high flow rate with different flow vectors: counterclockwise (P1) and clockwise (P3) from the bottom to top elevations.

In order to investigate the underlying process of a magnetically driven assembly under different flow conditions, we studied the real-time suspension flow patterns and calculated the effective flow rates and directional effective diffusion coefficients at different positions during film formation by using confocal laser scanning microscopy (CLSM) with fluorescent microbeads added to the CNC suspension (Figures 5, S17, and Table S1).^14^ It is worth noting that the microscopic flows observed with CLSM deviate relative to the overall direction due to Brownian motion but retain their general direction for a prolonged period (hours). To track fluid flow at three different layers within the CNC/MNP suspension (bottom, middle, top), fluorescent microbeads were added to the suspensions (Figures S17–S19 and Videos S1–S4). At P1, right-handed flow direction changes of fluorescent beads, 7 to 4’o clock, were observed along the layer from the bottom to the top as shown in Figures 5b, S18a and Video S1. The center area, P2, has a very slow flow rate that cannot influence the CNC alignment even though it has a minor right turn, as shown in Figures 5c, S18b and Video S2. At P3, the MNP aggregated area, the direction of fluid flows turns left-handed, clockwise, along the layer from the bottom to the top, as shown in Figures 5d, S18c and Video S3. The flow of the dried CNC/MNP suspension without a magnetic field is random and very slow (Figure S19 and Video S4). Further, the flow rate was quantified by using a diffusion coefficient equation for one-directional flow (Figure S20 and Table S1).

The overall summary of these observations is that the direction of flow within each layer rotates in a counterclockwise manner (top view), with the angular rotation reaching 70° from the bottom to the top layer. Furthermore, the flow rate in these magnetic gradients is 2–3 orders of magnitude higher than the flow rate during a regular assembly without a magnetic field, thus indicating the formation of intense local vortices from the rotational flow direction changes in contrast to very slow flow in suspensions drying without a magnetic field.

**Figure 6:**
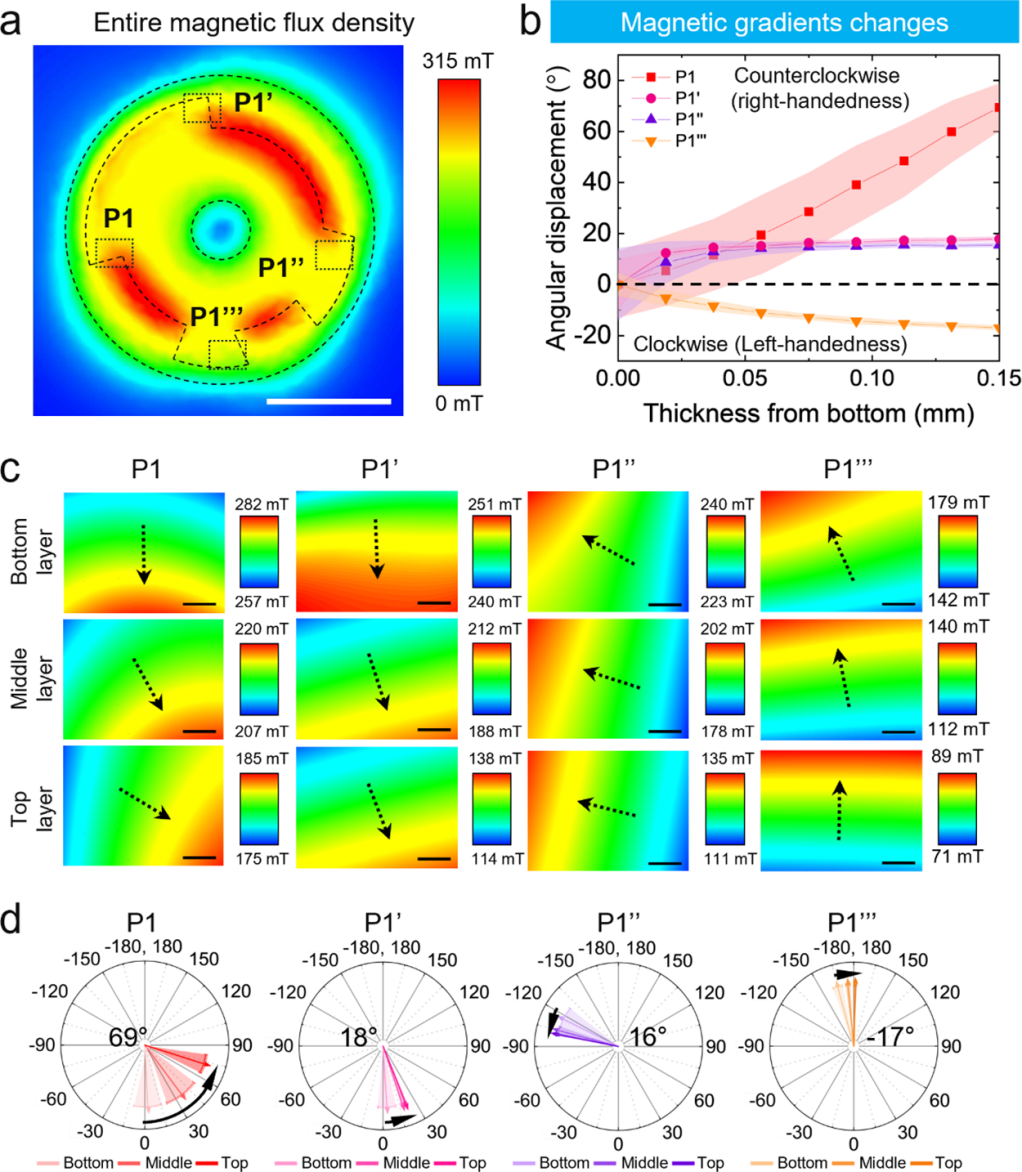
Simulated magnetic flux density for magnetic gradient change depending on the position. (a) Simulated entire magnetic flux density of the layer where the CNC/MNP film dried on the circularly patterned magnet. Scale bar is 1 cm. (b) Angular displacement of the magnetic flux density gradient at four different positions: P1, P1′, P1″, and P1‴. (c) Simulated local magnetic flux density gradient of four different positions, P1, P1′, P1″, and P1‴, with the bottom, middle, and top layers. The gap between the layer and the magnet is 1 mm. Scale bars represent 200 μm. (d) Direction changes of the average magnetic flux density gradient at four different positions, P1, P1′, P1″, and P1‴.

Therefore, we suggest that vortex-like local flow at a high flow rate can affect the twisted organization of assembled CNCs. In this case, counterclockwise directional vortices facilitate right-handed organization. To consider if local magnetic gradients can stimulate such flow within magnetic gaps, we simulated the magnetic flux density gradients in points of interest (Figures 6 and S21). The magnetic flux density simulation was implemented with nine sublayers including the same top, middle, and bottom layers, and the simulation results are enlarged at each position (P1, P1′, P1″, and P1‴) outside of the circular pattern to investigate local magnetic flux density gradients (Figure 6b,c).

To quantify the directional changes of magnetic flux density gradients, we measured the angles between three white vertical and red tangent lines at the boundary line that divides regions with identical magnetic flux density intersects (Figure S22). Three white lines divide four equally sized areas of simulated magnetic flux density for the equal distribution of crossed points. At the P1 area, the direction of the magnetic flux density gradient points toward the 6 o’clock direction at the bottom layer of P1. Then, at the middle layer of P1, the magnetic flux direction rotates 34° counterclockwise. Last, at the top layer of P1, the direction of magnetic flux density rotates 35° counterclockwise.

Overall, the magnetic flux density gradient along the bottom to the top gradually rotated in the counterclockwise direction, 69°, thus creating a local vertical vortex in the gap location (Figure 6c,d, **P1**). As the same sequences, the magnetic gradients rotate 18° and 16° to the counterclockwise direction at the P1′ and P1″ areas, while the P1‴ area has a clockwise directional rotation by 17° (Figure 6c,d, **P1′**, **P1″**, **P1‴**). As expected, the P2 area, the center of the film, has a negligible gradient under 5 mT. From prior results,^14^ the minimum magnetic gradient needed to generate directional flow should be higher than 7 mT (Figure S23).

**Figure 7:**
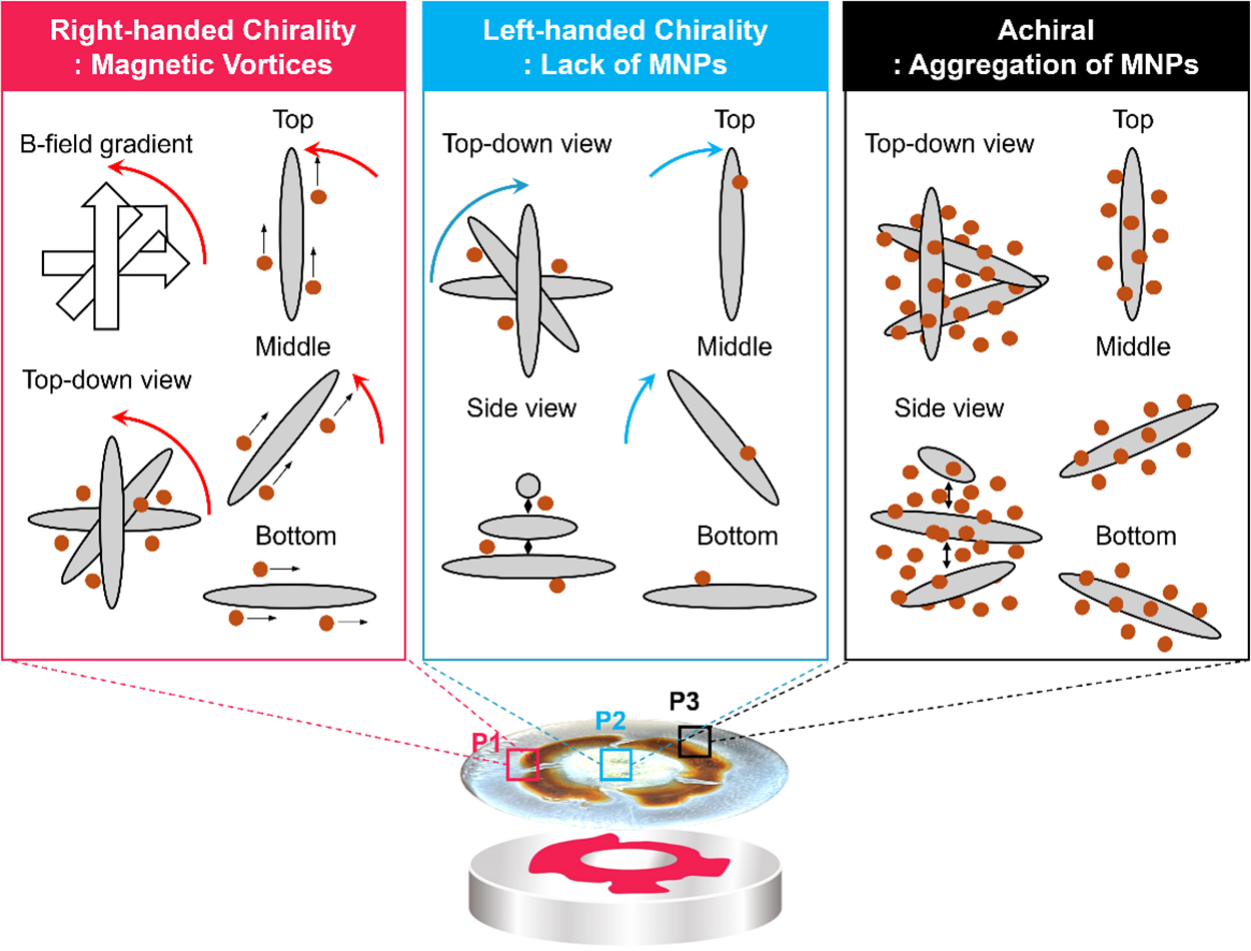
Schematic for the localized magnetic field-induced chirality alternation of CNCs. At P1, CNCs are organized into right-handed structures due to the rotation direction change of the magnetic gradient from the bottom to the top. At P2, CNCs are self-assembled into left-handed structures as pristine CNCs due to the small amount of MNPs. At P3, CNCs have achiral structures due to excessive MNP aggregation.

Overall, a change in the gradient direction of the magnetic flux density was observed in a counterclockwise, right-handed direction from the bottom to the top layer at different locations. This change forms vortices with different strengths and rotation directions, thus facilitating different helical organizations for different locations, as suggested by the complementary experimental methods and illustrated in Figure 7.

In these schematics, we suggest that the clockwise and counterclockwise twisting of magnetic CNCs within localized gaps with clockwise and counterclockwise magnetic gradients defines the highly localized appearance of left- and right-handed chiro-optical characteristics. Indeed, in our very recent work, we demonstrated inverse chiral optical appearance on large-scale preprogrammed aligned thin films by using shear-induced printing of birefringent CNCs with a controlled twisting angle of stacked nanolayers with a unidirectional orientation.^18^ These clockwise and counterclockwise printed twisted structures showed inversed symmetrical CD signals. This inversion was simulated with FDFT modeling of stacked anisotropic nanolayers that confirmed the direct relationship between the twisting vector direction and the appearance of left- or right-handed chiroptical signals.

## Conclusions

In conclusion, in this study, we demonstrated the local chirality patterning of thin films made from magnetic polysaccharide (cellulose) nanocrystals during the evaporation-induced assembly within patterned magnetic fields with narrow magnetic gaps. This local chirality change from natural left- to induced right-handedness is facilitated by the formation of local flow vortices with different angular rotation gradients within magnetic gaps in different magnetic pattern locations. As we suggested, these evaporation-/field-induced localized flow vortices with different rotations from the bottom to the top can control the helical organization of magnetically decorated CNCs and trigger chiral inversion during the confined flow of suspensions and can be fixed after complete drying.

The localized inversion in the handedness of twisted organizations induced by flow-induced rotations of the needle-like nanocrystals within local magnetic vortices originated from the interplay between the direction and strength of the complex magnetic field gradients within the narrow gaps and evaporation-driven flows of liquid crystalline suspensions at the receding front. We suggest that this observation will be important for the future development of patterned twisted optical metamaterials to achieve artificial magnetically tailored patterned photonic crystals with tunable chiroptical properties for prospective optical communication, chiral nonlinear photonics, induced photoluminescence, lasing, or enantiomeric sensing.^11,34^

## Methods

### Preparation of CNCs

CNCs were prepared by sulfuric acid hydrolysis of wood pulp according to an established protocol.^35^ After rinsing and drying the pulp, 17 g of dried wood pulp was dispersed into 300 mL of 64 wt % sulfuric acid (aqueous) at 45 °C and mixed using a magnetic stirrer for 60 min. The reaction was stopped by adding the suspension to deionized water, which is 10-fold the amount of the suspension. After phase separation occurred overnight, the bottom layer was concentrated by centrifugation twice with a large excess of Nanopure water. The suspension was purified using dialysis membranes in deionized water for a minimum of 4 days with a daily exchange of water until the pH value of the water outside of the dialysis membrane became constant. To ensure homogeneity and satisfactory dispersion, the suspension was centrifuged twice more and tip-sonicated. The CNC suspension was stored at room temperature.

### Synthesis of MNPs

MNPs were synthesized according to an established method.^25^ Under a N_2_ atmosphere and at 70 °C, a round-bottom flask was charged with FeCl_2_·4H_2_O (5 mmol) and FeCl_3_·6H_2_O (10 mmol) into 50 mL of MilliQ water and stirred for 30 m. 6 mL of ammonium hydroxide NH_3_(aq) was added, and the system was stirred for another 30 min. A solid citric acid solution (1.5 g) was added to the system, and the mixture was stirred for 120 min under a N_2_ atmosphere and at 70 °C. The resulting solution was cooled to room temperature and then washed with MilliQ water and filtered by centrifugation three times (10,000 rpm, 10 min round). Finally, the solution was dialyzed for 72 h against deionized water. From TEM images, the average size of MNPs was ∼7.0 nm (Figure S1).

### Film Casting from CNC/MNP Suspensions

An aqueous 0.25 wt % Fe_3_O_4_ MNP solution is added by weight to a 1 wt % CNC suspension (wt ratio between CNC and MNP is 69:1). The CNC/Fe_3_O_4_ suspension is sonicated for homogeneity for 30 s (5 s on, 5 s off) at 40% amplitude by tip sonication, followed by vigorous stirring of the mixture for 24 h at 24 °C. The films are produced via EISA after drop-casting the suspension in a 35 mm diameter Petri dish.

### Magnets and Processes

Magnets were chosen based on commercial availability to demonstrate affordability of the method. Commercially available magnets from Amazon (coin-shaped, diameter 32 mm × height 2 mm, 150 mT field strength) and McMaster were purchased. The patterned magnetic fields employed were from Polymagnets from Correlated Magnetics.^36^ The customized magnets have commercial applications that can impart distinctive patterns into these composite thin films. Magnets were placed directly under the Petri dishes during EISA film production. The strength of the magnetic field for the magnet was measured using a Gaussmeter (TD8620, VETUS Industrial Co.) that can measure the surface flux density of permanent NdFeB magnets with a measurement accuracy of ±5%. The shape of the magnetic field was photographed with a magnetic viewing film. Magnetic viewing films are plastic films coated with a slurry of nickel nanoparticles, used to visualize magnetic fields or magnetic tapes from CMS Magnetics.^37^ Nonuniform field distribution from the Polymagnet magnets was not measured with the Gaussmeter due to sensitivity limitations.

### Dynamic Light Scattering (DLS)

DLS to measure the hydrodynamic diameter was conducted using a Zetasizer (Nano ZS, Malvern). The MNP suspension was diluted 100 times (0.0025 wt %) and sonicated before conducting DLS in a disposable plastic cuvette.

### Optical Microscopy

Optical microscopy images were collected with an Olympus BX51. CLSM was conducted under 500 nm (120Q, Lumen Dynamics) using an optical microscope (BX51, Olympus) with a ×10 objective.

### Transmission Electron Microscopy (TEM)

TEM was performed using a Hitachi HT7700 microscope to measure the diameter of MNPs with the help of ImageJ software. The MNP suspension was diluted to 1/10, dropped on the carbon TEM grid, and dried under an ambient state.

### CD and Ultraviolet–Visible (UV–Vis) Transmission Spectroscopy

CD and UV–vis transmission spectroscopy were performed using a Jasco J-815 CD spectropolarimeter with a dried sample film mounted perpendicular to the beam path (Figure S4). To measure the CD at local points, the part of the film with the region of interest was detached from the Petri dish. The dimension of the film was 1.2 mm × 1.2 mm or 13 × 22 μm (CNC-rich area and MNP-rich area, respectively). After that, the detached dried sample was sandwiched between 1.25 cm × 1 cm quartz slides and mounted on the sample holder (Figure S4a).^38^ Then, CD and UV–vis transmission spectroscopy were performed with a sample holder (Figure S4b). The specific measurement condition was as follows: measurement range: 200–800 nm; bandwidth: 1 nm; data pitch: 1 nm; and scanning speed: 100 nm/min. For the reproducible CD signals, the samples are rotated at 45°.

### High-Resolution Scanning Electron Microscope (HR-SEM)

HR-SEM micrographs were obtained with Hitachi SEM instruments (SU8230) by straight cutting samples and sputter-coating with a thin layer of gold (∼4 nm thickness).

### Atomic Force Microscopy (AFM)

AFM images were obtained using a Bruker Dimension Icon in the standard tapping mode in air.^39^ Scans of the films are conducted with a regular tip of ∼8 nm radius, 1:1 ratio, and 512 pixels.

### XPS

XPS was conducted with a fully dried CNC/MNP film using Thermo K-Alpha XPS. The three different local positions, P1, P2, and P3, were measured using an optical microscope to ensure precise positions of the focused beam. To confirm the relative amount of MNPs at the local position, the binding energy between 700 and 740 eV was enlarged to observe distinct peaks at 711 and 724 eV, which correspond to Fe 2p 3/2 and Fe 2p 1/2 from Fe_3_O_4_, respectively.^40^

### Magnetic Field Simulation

Simulation of magnetic flux density was conducted by Ansys Maxwell (Figures 6 and S21-S23). The patterned magnet was divided into three regions, a circular center, an area between the center circle and the patterned boundary, and an outer area. Each area has a different magnetic pole based on the measurement by a Gaussmeter. The shape of each area was designed based on Figure 2d. The N35-grade NdFeB magnets are selected for the simulation. The layer was divided into nine layers with the same depth to explore the changes in magnetic field gradients.

### Flow Tracking

To track the fluid flow of the CNC/MNP solution, 1 μL of aqueous fluorescent microbeads (Latex beads, carboxylate-modified polystyrene, fluorescent yellow-green, Sigma-Aldrich) was added to the CNC/MNP solution before a mixing procedure, which is mentioned in the preparation section. After mixing, 4 mL of solution was deposited into the circular Petri dish (35 × 5 mm) on the patterned magnet. The deposited solution was evaporated under 10% relative humidity at 22 °C, and the fluid flow was recorded by CLSM when the depth of the solution was lowered to about one-third of the initial depth (5 mm), which took about 9 h. The total measuring time was 10 min. In the case of the top layer at P3, the total measuring time was 5 min since the movement of fluorescent beads in the measuring screen was occupied from the start to the end point of the measuring screen in 5 min. The real-time particle tracking was conducted by an open-source software (Tracker 6.0.1).^41^

### Calculation of *g*-Factor

The *g*-factor, which quantifies the chirality strength, was calculated from the CD (θ) and UV–vis absorbance (*A*) using the following equation^31^:
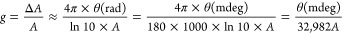


Both CD spectra and light absorbance were measured by a Jasco J-815 CD spectrometer.

### Mueller Matrix Spectroscopy

The spectroscopic ellipsometer Woollam M-2000U with VWASE software was utilized for the measurement of optical activities in the 245–1000 nm wavelength range. The laser beam size of the ellipsometer was 3 × 3 mm, and the film was cut into 2 × 2 mm pieces representing different locations. The samples were sandwiched between two quartz slides with sizes of 1 cm × 1 cm × 1 mm and mounted onto the holder. Then, the measurements were repeated 100 times to increase the signal-to-noise ratio. The m_14_ component, which corresponds to a CD contribution, was obtained in the transmission mode with MM component analysis normalized with respect to *m*_11_.^42^

## Supporting Information Available

TEM images of MNPs and size distribution, hydrodynamic diameter distribution of MNPs, photographs of films with/without MNPs and pattern of magnets, photograph of the CD measurement setup, photographs of CNC/MNP composites evaporated on the various patterned magnets with CD, UV–vis spectra of CNC/MNP composites, Mueller matrices at each P1 position, calculated depolarization index and equation, direct comparison between commercial CD and CD elements from the Mueller matrix, optical micrographs and AFM images of CNC/MNP composites, XPS of CNC/MNP composites at each P1 position, trajectory of fluorescent microbeads and their diffusion coefficients, simulated magnetic flux density of the P2 position and guidelines to measure the direction of gradients, simulated magnetic flux density of the coin-shaped magnet, and table of diffusion coefficients (PDF)
CLSM of the CNC/MNP suspension on the patterned magnet at P1 with top, middle,and bottom layers (MP4)
CLSM of the CNC/MNP suspension on the patterned magnet at P2 with top, middle,and bottom layers (MP4)
CLSM of the CNC/MNP suspension on the patterned magnet at P3 with top, middle,and bottom layers (MP4)
CLSM of the CNC/MNP suspension in the absence of a magnet under an ambient state with top,middle, and bottom layers (MP4)
