# Supplementary material for: Magneto-Responsive Chiral Optical Materials: Flow-Induced Twisting of Cellulose Nanocrystals in Patterned Magnetic Fields
Source: ACS Nano. 2024 Sep 5;18(37):25512–21. doi: 10.1021/acsnano.4c05320 (PMC11411705; doi:10.1021/acsnano.4c05320)
Supplement: Supplementary file 1 — nn4c05320_si_001.pdf [file nn4c05320_si_001.pdf]

Supporting Information

Magneto-Responsive Chiral Optical Materials: Flow-Induced Twisting of Cellulose Nanocrystals in Patterned Magnetic Fields

*Minkyu Kim[1,2+], Jisoo Jeon[1+], Kellina Pierce[1], Daria Bukharina[1], Woosung Choi[1], Jinyoung Choi[1], Dhriti Nepal[3], Michael E. McConney[3], Timothy J. Bunning[3], Vladimir V. Tsukruk[1] **

[1] School of Materials Science and Engineering, Georgia Institute of Technology, Atlanta, GA 30332, USA

[2] Department of Chemical Engineering, Dankook University, Yongin 16890, Republic of Korea

[3] Air Force Research Laboratory, Wright-Patterson Air Force Base, Ohio 45433, USA

[+]These authors have equal contributions to this paper.

*Corresponding Author E-mail: vladimir@mse.gatech.edu, Phone: 404-894-6081.

D. Nepal, M. McConney, T. J. Bunning,

Air Force Research Laboratory, Wright-Patterson Air Force Base, Ohio 45433, USA

Video S1. CLSM of the CNC/MNP suspension on the patterned magnet at P1 with top, middle, and bottom layers. The rate of the video accelerated 30x.

Video S2. CLSM of the CNC/MNP suspension on the patterned magnet at P2 with top, middle, and bottom layers. The rate of the video accelerated 30x.

Video S3. CLSM of the CNC/MNP suspension on the patterned magnet at P3 with top, middle, and bottom layers. The rate of the video accelerated 30x.

Video S4. CLSM of the CNC/MNP suspension in absense of magnet under ambient state with top, middle, and bottom layers. The rate of the video accelerated 30x.

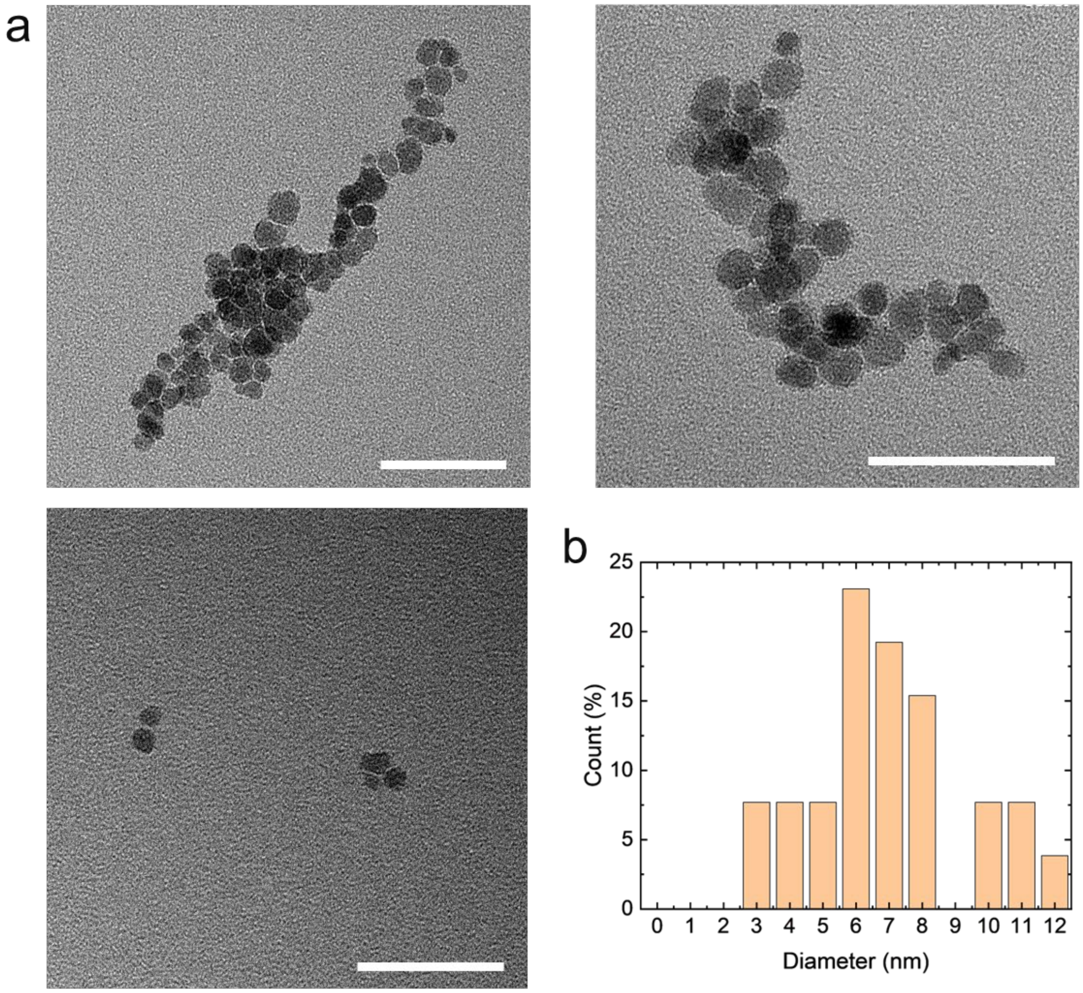

Figure S1. (a) TEM images of MNPs and **(b)** size distribution of MNPs. The mean diameter of MNPs was measured from TEM as 7.0 ± 2.3 nm. Scale bars: 50 nm.

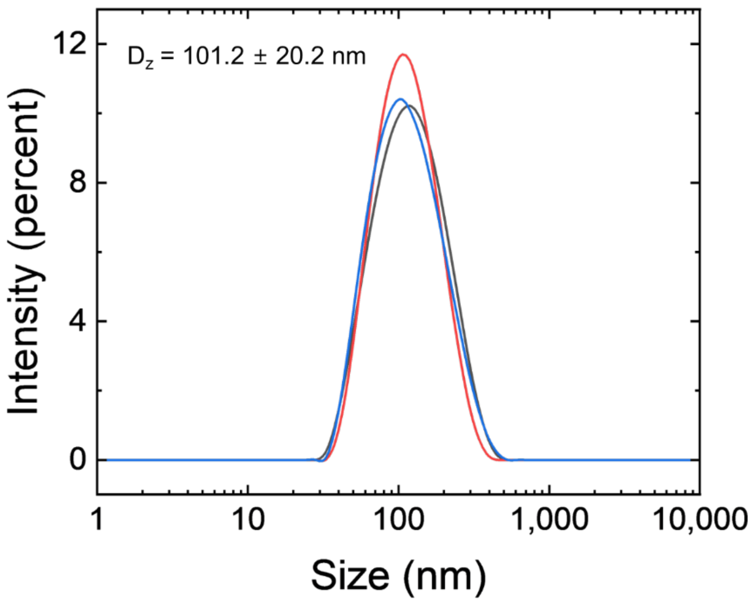

Figure S2. Hydrodynamic diameter distribution of MNPs from DLS. Z-average diameter is 101.2 nm.

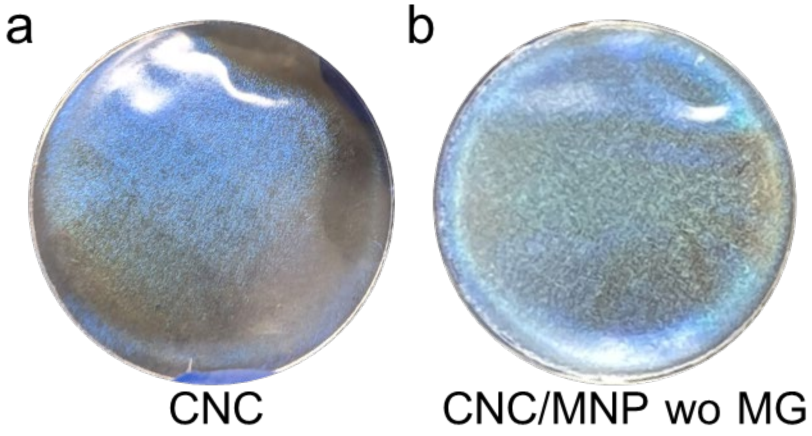

Figure S3. Photograph of (**a**) CNC and (**b**) CNC/MNP films dried without magnet.

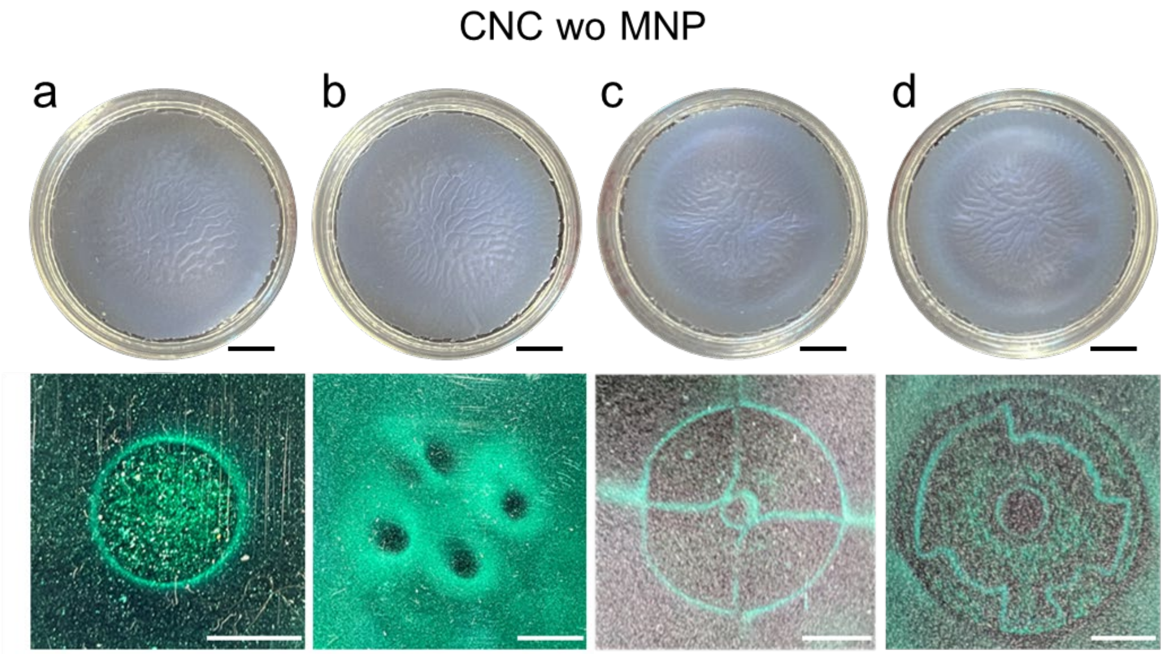

Figure S4. Photographs of pure CNC film dried under different patterned magnetic fields: (**a**) coin, (**b**) four dots, (**c**) cross, and (**d**) circular shaped magnetic pattern as the control of the CNC/MNP films dried under patterned magnetic fields presented in **Figures 2a-d**. Scale bars: 1 cm.

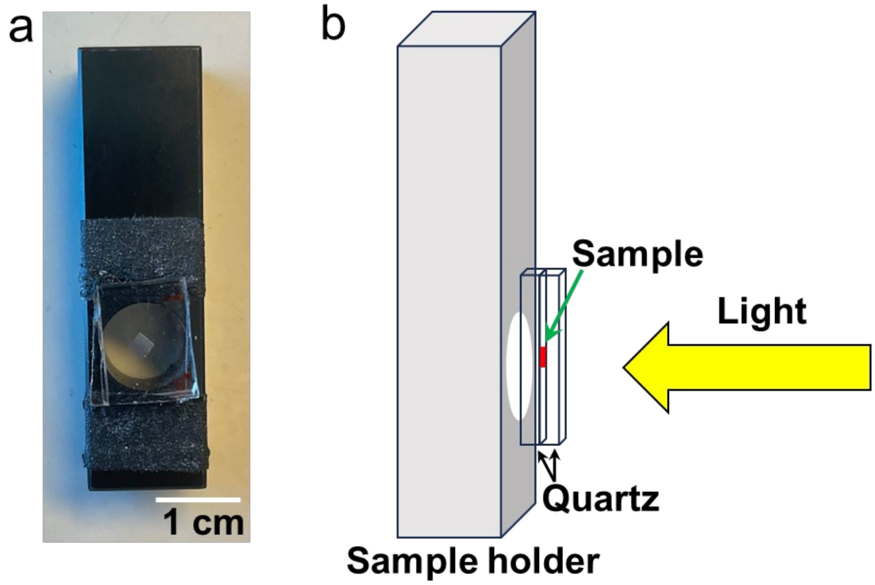

Figure S5. (a) Photograph of sample sandwiched between quart glasses on the sample mount holder of CD machine and (b) scheme of CD measurement of the sample.

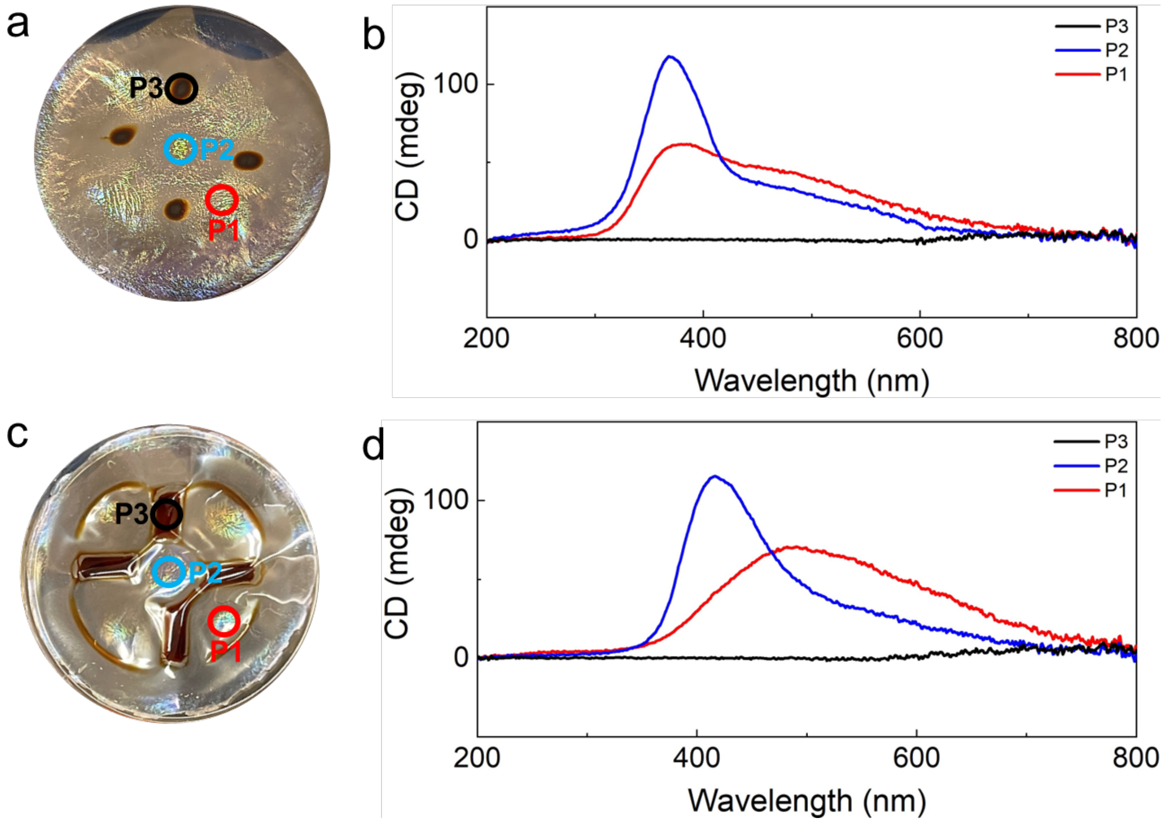

Figure S6. Photograph of CNC/MNP composites films evaporated on a (**a**) four dots-patterned magnet and (**c**) a cross patterned magnet with selected three position. Scale bars: 1 cm. The CD spectra of CNC/MNP film evaporated on a (**b**) four dots patterned magnet and (**d**) a cross patterned magnet depending on the position of the films.

These changes can be related to the variability of magnetic flux within different gaps as was shown in simulations discussed below. For the composite films dried on four dots and cross-shaped magnets, they did not show the chirality inversion (**Figure S6**). In comparison with the circularly patterned magnet, the other magnets do not have the asymmetric corners, that make asymmetric gradient direction changes of magnetic field. Coin-shape magnet and dot magnets have only linear and radial magnetic fields patterns that only generate unidirectional flows (**Figure S6**).

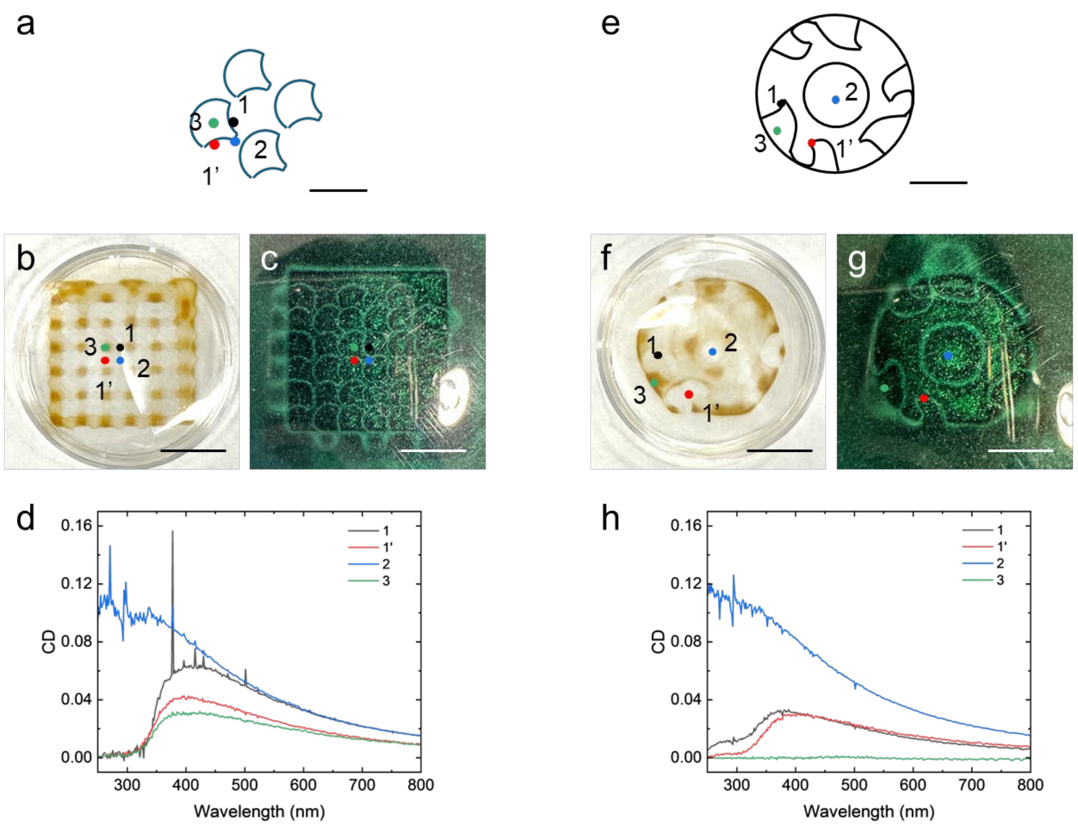

Figure S7. (a, e) Magnetic pattern of permanent magnets. (b, f) Image of CNC/MNP films evaporated on the patterned magnets. (c, g) magnetic patterns through the magnetic viewing film. (d, h) CD signals of each CNC/MNP film from Mueller matrix. Scale bars; (a) 5 mm, (b, c, e, f, g) 1 cm.

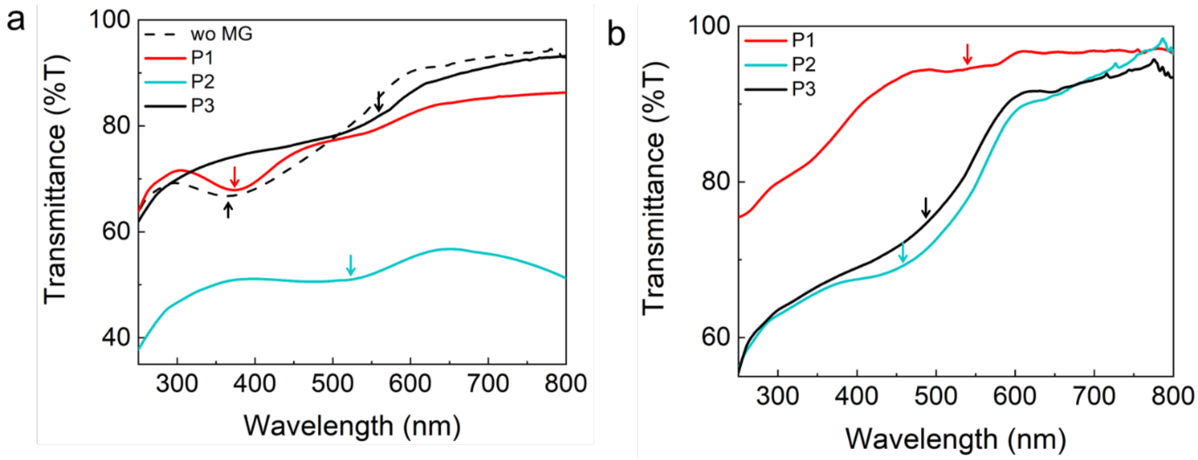

Figure S8. UV-vis spectra of the of CNC/MNP composites films evaporated on a (**a**) coin shape magnet and (**b**) a circular patterned magnet. The UV-vis spectrum of the composite film dried without magnetic field also plotted as a control in (**a**).

Figure S8a illustrates that the reflectance peak of the composite film dried without magnet and dried under coin magnet at P1-3 are matched with CD peak at **Figure 3b**. This result indicates that the red shift at the P2 and P3 are due to the increased pitch distance from the concentrated MNP at the positions. **Figure S8b** exhibits that reflectance peak of the composite film dried under circular patterned magnet at P1 and P2 are matched with CD peak at **Figure 3d**. This result shows that P1 selectively reflect the right-handed circularly polarized right whereas P2 selectively reflect the left-handed circularly polarized right. Despite P3 possesses weak reflectance peak at ~500 nm as presented in **Figure S8b**, it cannot show the CD peak because chiral structure is not formed at P3 position at shown in **Figure 2d**.

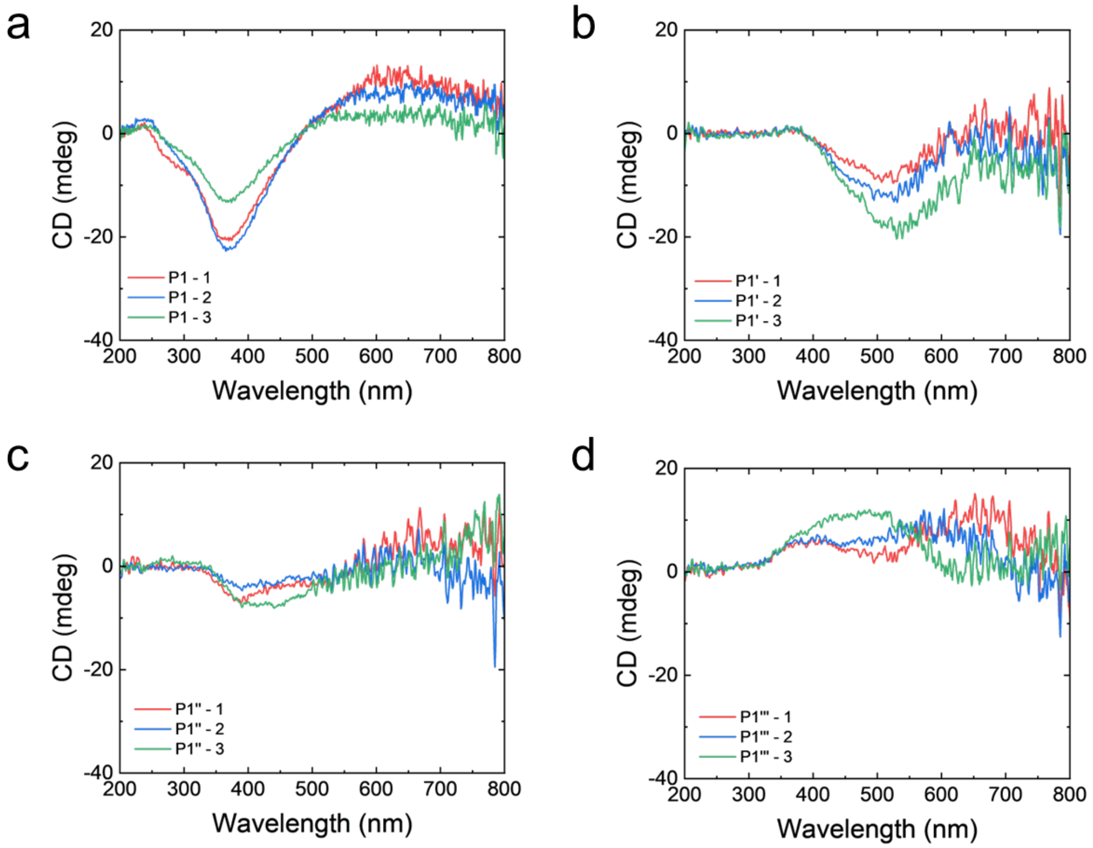

Figure S9. CD spectra of multiple samples at (a) P1, (b) P1’, (c) P1’’, and (d) P1’’’.

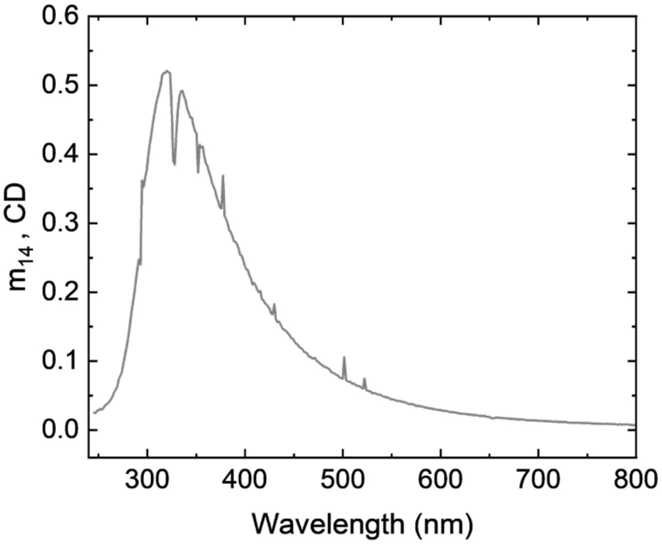

Figure S10. m_{14}, CD component, of CNC film measured from Mueller matrix analysis with spectroscopic ellipsometry.

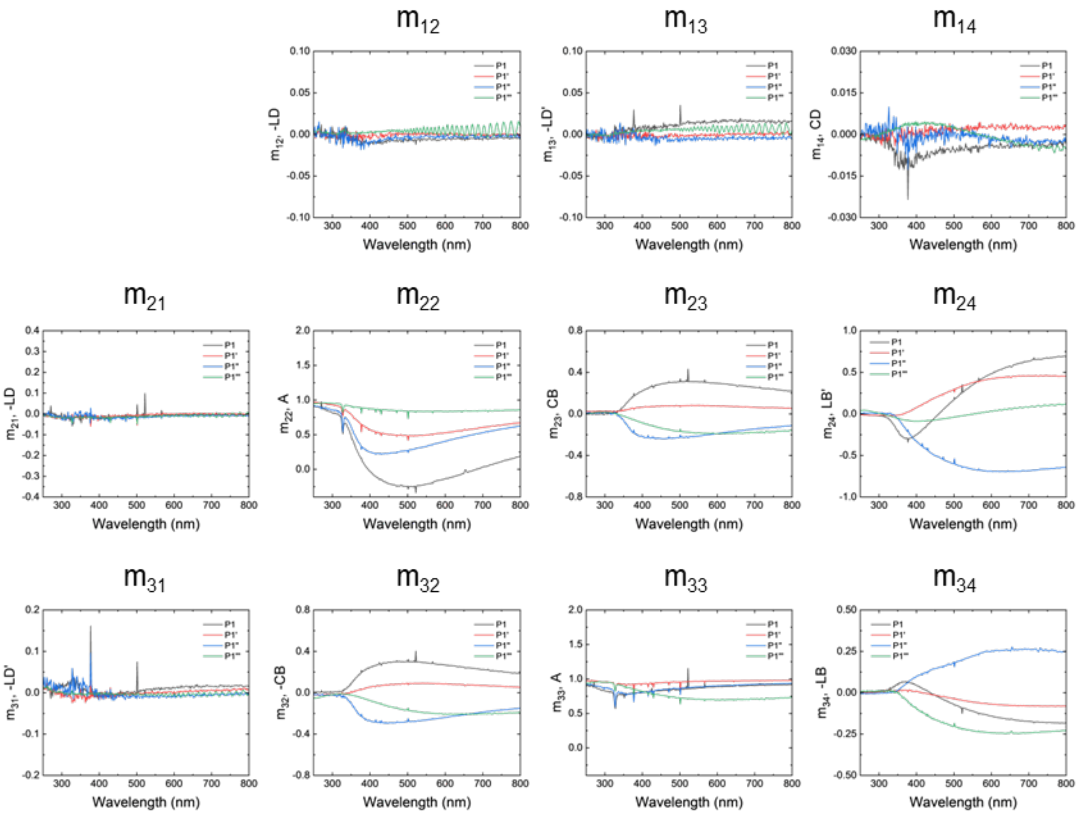

Figure S11. All measured Mueller matrix elements in each location.

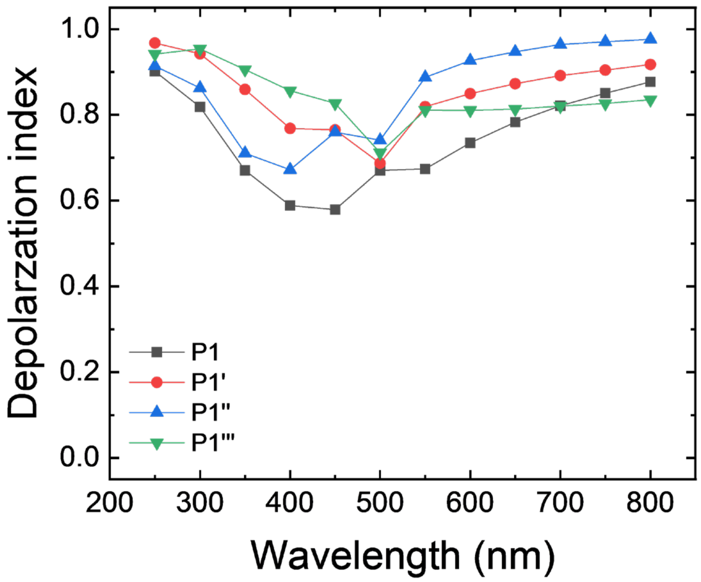

Figure S12. Calculated depolarization index (DI) of each Muller matrix.

"For non-depolarizing samples, the Muller matrix can be circular dichroism/birefringence (CD and CB), and linear dichroism/birefringence (LD and LB) as shown below matrix.

$$\begin{bmatrix} A & -LD & -LD' & CD \\ -LD & A & CB & LB' \\ -LD' & -CB & A & -LB \\ CD & -LB' & LB & A \end{bmatrix}$$

Depolarization index (DI) was calculated by the following equation.

$$DI\ [M] = \frac{\left(\sum_{i,j=0}^{3} M_{i,j}^{2} - M_{0,0}^{2}\right)^{1/2}}{\sqrt{3}M_{0,0}}$$

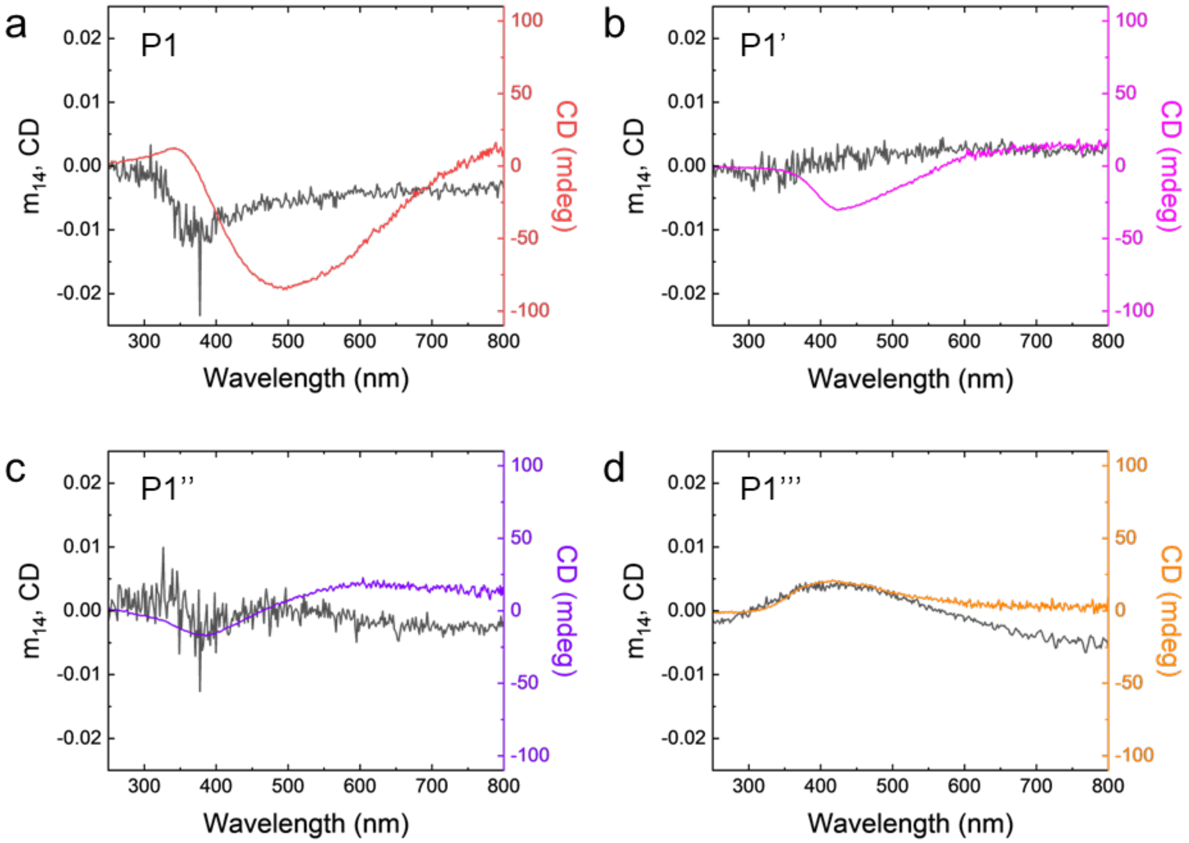

Figure S13. Direct comparison of CD signals at each P1 position from commercial CD equipment and Muller matrix analysis of spectroscopic ellipsometry.

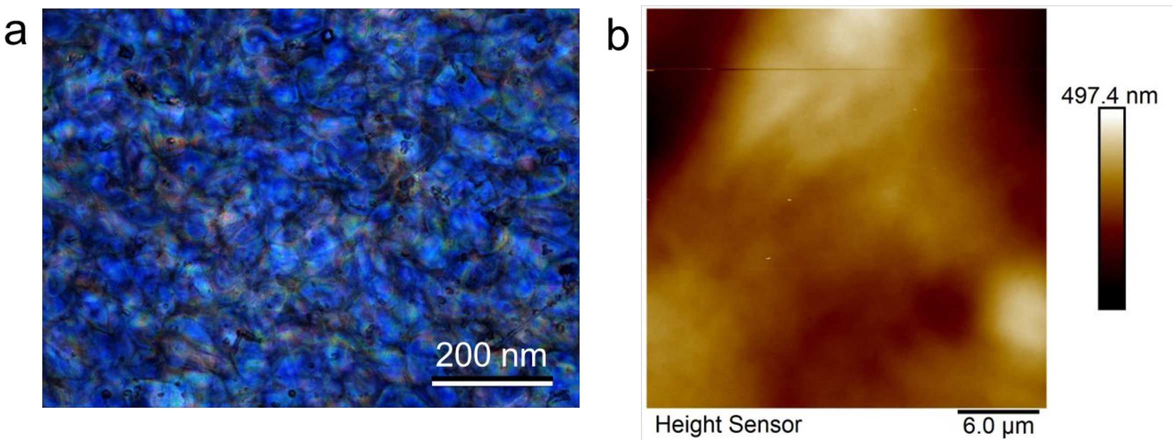

Figure S14. (**a**) OM and (**b**) AFM image of CNC/MNP composite film dried without magnet field, prepared as a control sample to CNC/MNP composite film dried under the magnetic fields.

Partial separation between CNC suspension and MNPs results in free MNPs aggregation along the circumstance of the Petri dish with decorated CNCs following the flow pattern across the rest of the area (**Figure S15**). The MNPs are moved to a region with high magnetic flux density that forms the partial separation between MNPs and CNC suspensions within a few hours.

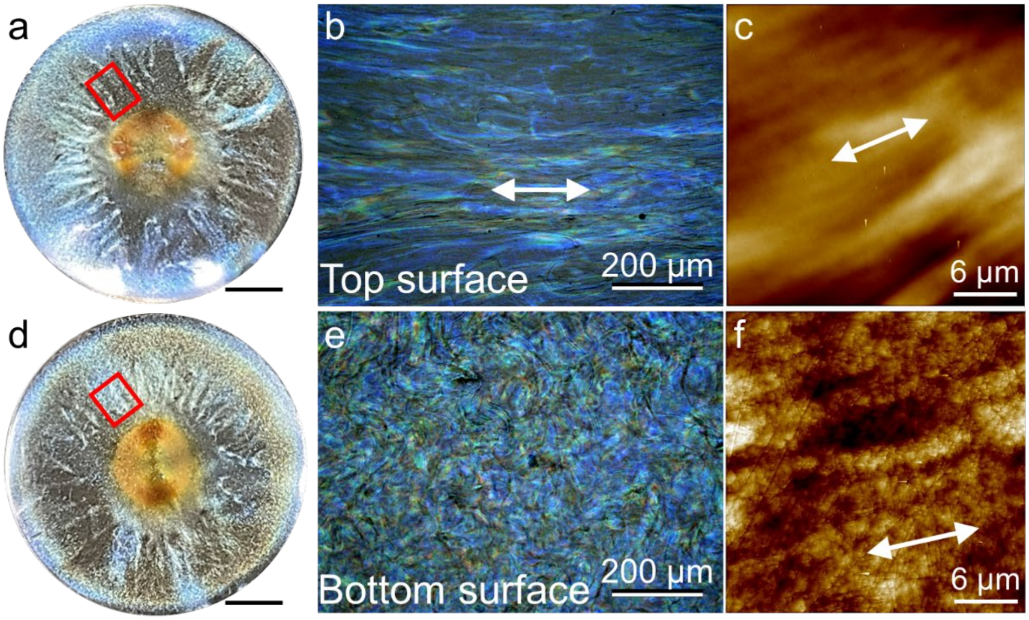

Figure S15. (**a**, **d**) Photograph, (**b**, **e**) optical microscopy, (**c**, **f**) topography AFM image of CNC/MNP film dried on coin shaped magnet: (**a**-**c**) top surface of the film and (**d**-**f**) bottom surface of the film.

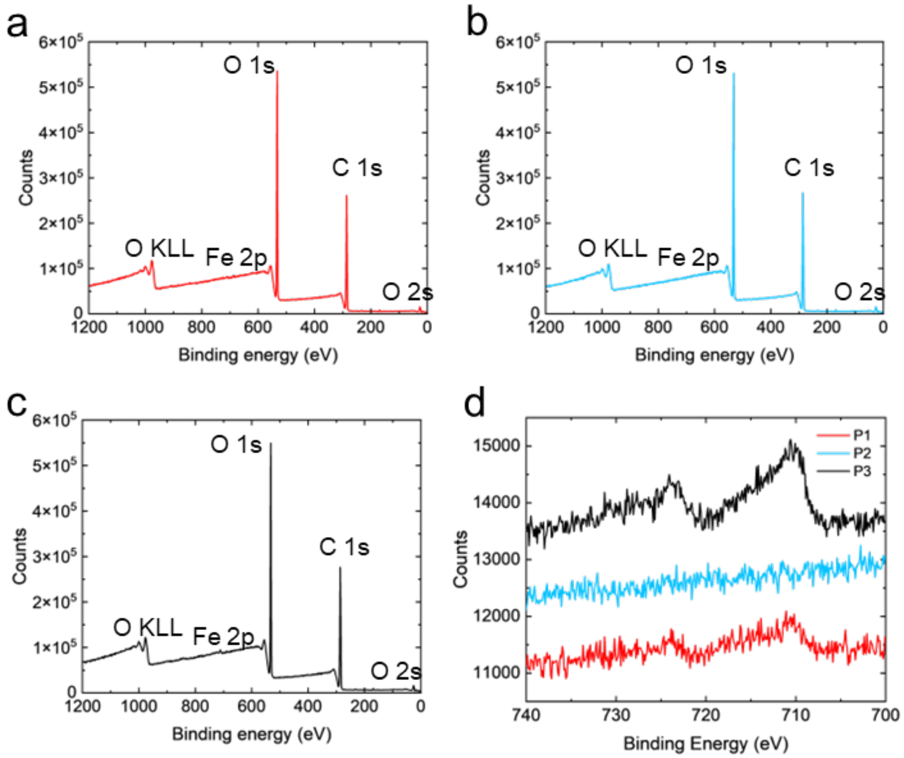

Figure S16. XPS measurement of CNC/MNP film at each position. (a) P1, (b) P2, and (c) P3. (d) Fe 2p signals at each position.

X-ray photoelectron spectroscopy (XPS) was conducted to qualitatively confirm existence and amounts of MNPs in the P1, P2, and P3 area (**Figure S16**). Distinct peaks at 711 eV and 724 ev, which corresponds to reported peaks for Fe 2p 3/2 and Fe 2p 1/2 from Fe_3O_4, respectively, were observed for P1 and P3. While it can be assumed that the chemical conformation of the Fe elements is basically same for the P1 and P3 from the unchanged peak locations for both points, the difference in height of the peaks, in which P3 area displays the much stronger signals of Fe 2p compared to P1, also shows that the concentration of the NMP is higher in P3 region. In contrast, no significant peaks were observed in the region from the measurement conducted at P2, indicating that the P2 area contains negligible amount of Fe_3O_4 MNPs.

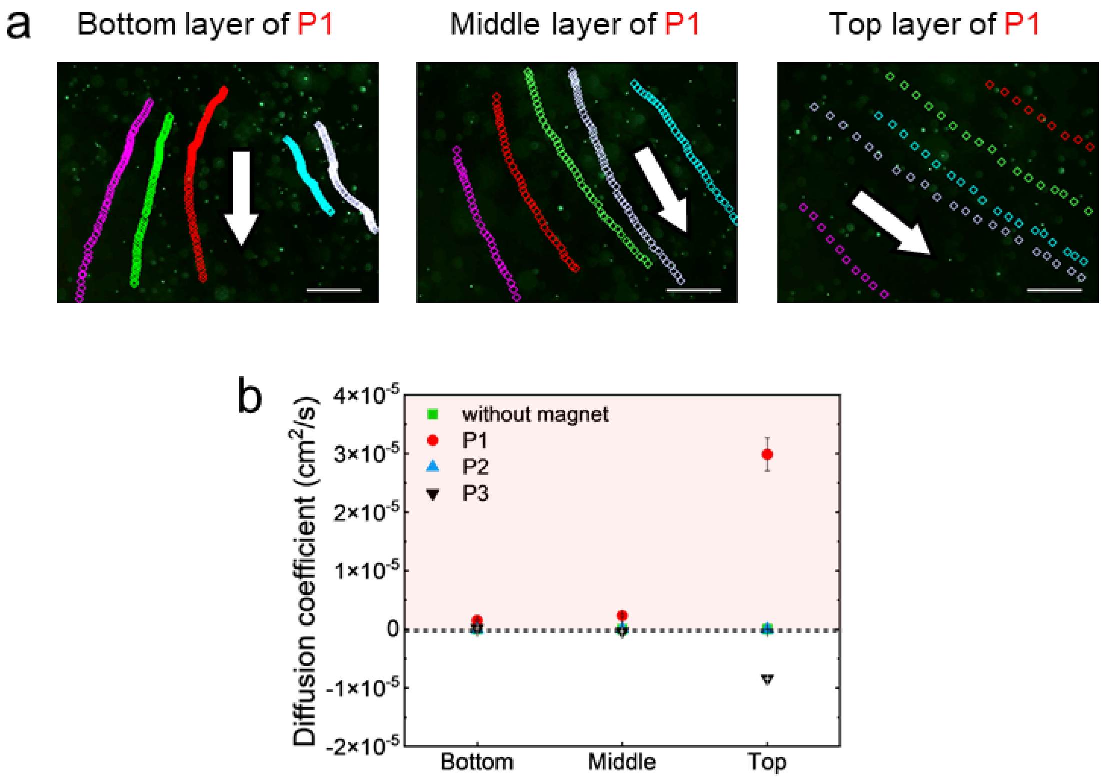

Figure S17. (**a**) Resulting optical images with tracking marks of fluorescent microbeads at bottom, middle, and top layer at P1 position. Time duration in the graph: 10 min (bottom layer), 3 min (middle layer), and 1 min 30 sec (top layer). (**b**) Diffusion coefficients of fluorescent microbeads at P1-3 at bottom, middle, and top layer under magnetic field and without magnet (derived from Figures S18, 19).

Overall, random directional changes observed for different batches at later stages can be related to changing suspension concentration and viscosity during evaporation and results discussed are relevant to mid-stages of film forming.

We explored flow pattern at three positions (P1-3) and the P1- P1‴ area is selected as representative example for detailed study. The suspension is divided into bottom, middle, and top layers, and the gap between each layer is about 0.75 mm as depicted in **Figure 5a.** The CNC/MNP suspension dried about 1/3 of initial solution amount to concentrate the suspension over 3 wt.% to explore the flow within the nematic phase.

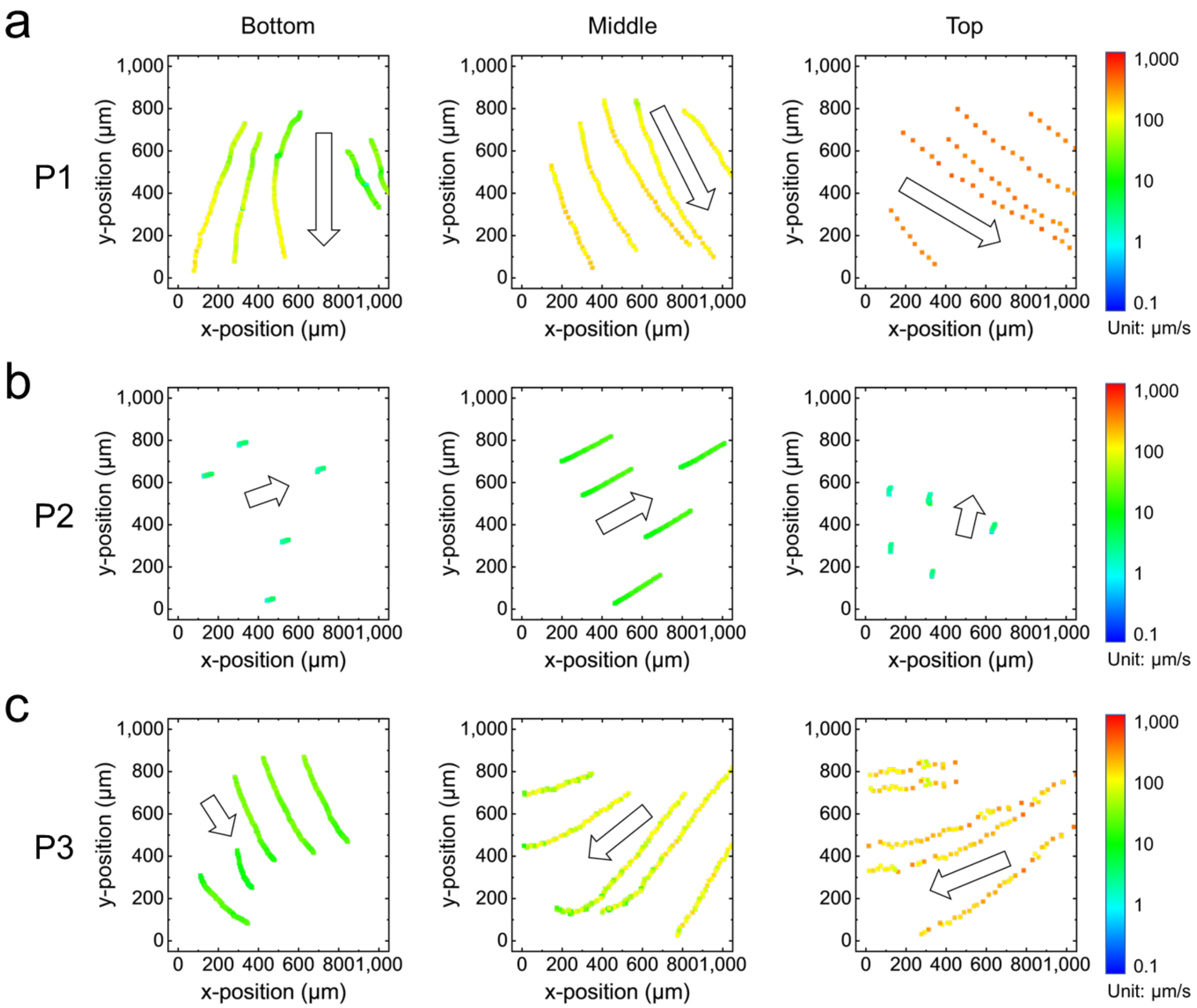

Figure S18. Fluorescent microparticles trajectories of the CNC/MNP suspension on the circular patterned magnet at each position (a: P1, b: P2, c: P3). Arrows indicate the direction of flows in each layer. Time increments are (a, c) 5 s and (b) 20 s.

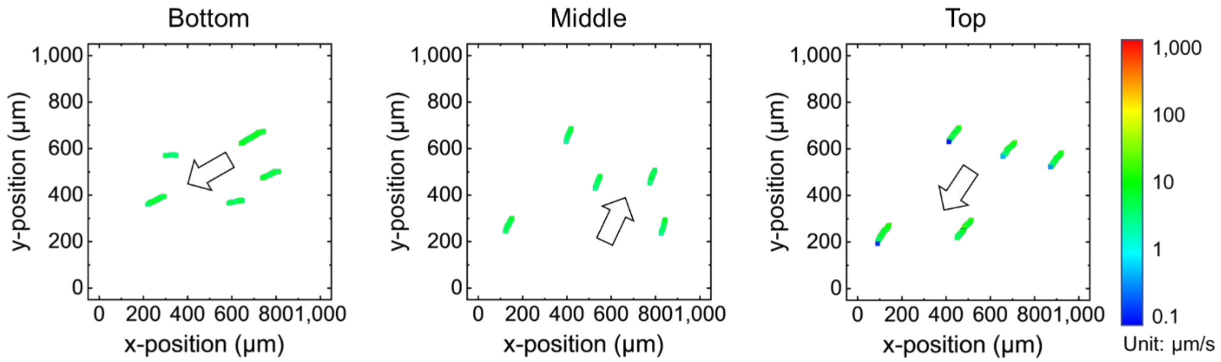

Figure S19. Fluorescent microparticles trajectories of the CNC/MNP suspension in absence of magnet field under ambient state. Hollow arrows indicate the direction of flows in each layer. Arrows indicate the direction of flows. Time increment is 5 s.

Details of CLSM

At P1, outside of circular pattern right-handed flow direction changes of fluorescent beads was observed along the layer from bottom to the top as shown in **Figures S17, S18a** and **Video S1**. In detail, at the bottom layer of P1, the fluorescent beads move downward between 5 to 7 o'clock direction. Then, at the middle layer of P1, the fluorescent beads move in the 5 o'clock direction. Last, at the top layer of P1, the fluorescent beads move in the 4 o'clock direction. Overall, the change in flow pattern was observed in a counterclockwise direction, right-handed direction, from the flow in the 7 o'clock direction at the bottom layer to the particle flow in the 4 o'clock direction on the top layer (**Figure S17** and **S18**). The center area, P2, has a very slow flow rate that cannot influence the CNC alignment even though it has a minor right turn as shown in **Figure S18b** and **Video S2**. The fluid flows directed to 2 o'clock at both bottom and middle layer, then the fluid at top layer flows to 1 o'clock.

At the P3, MNP aggregated area, the direction of fluid flows turns left-handed, clockwise, along the layer from bottom to the top as shown in **Figure S18c** and **Video S3**. The fluid at bottom layer directed to 5 o'clock along the MNP line direction. At the middle and top layers, the fluid flows to 7 and 8 o'clock direction. Even though magnetic gradient-induced flows occur in this area, large amounts of aggregated MNPs disrupt the evaporation-driven assembly of CNCs. As shown in **Figures 2f** and **2i,** there are a lot of achiral MNPs accumulated in MNP line area, P3.

To quantify the effects of magnetic gradient induced flow on the alignment, a diffusion coefficient of fluorescent microbeads (D) was calculated at each position from the Einstein equation: $D = \overline{\Delta^2}/2t$ the $\overline{\Delta^2}$ is the mean square of the deviation and t is the time of observation (**Figure S20, Table S1**).

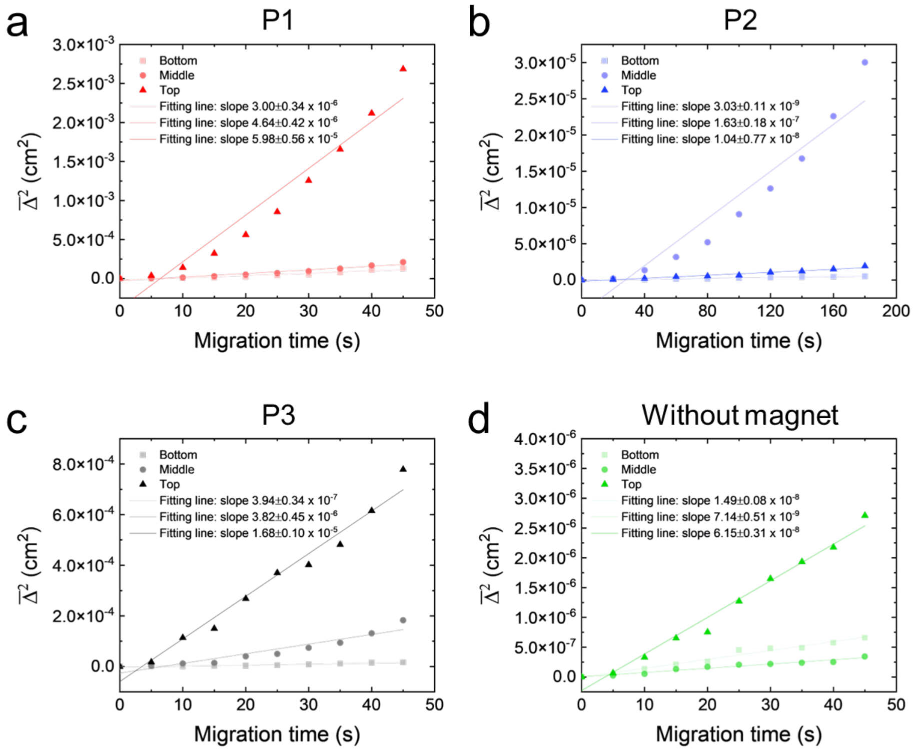

Figure S20. Mean square deviation vs diffusion time graphs for the magnetic gradient-induced flow at each position. a: P1, b: P2, c: P3, and d: without magnet.

The slope of the linearly fitted regressions is equal to two times of the diffusion coefficient (D). The D at P1 is higher than the Ds at P3 and the freely dried CNC/MNP suspension about two-three orders.

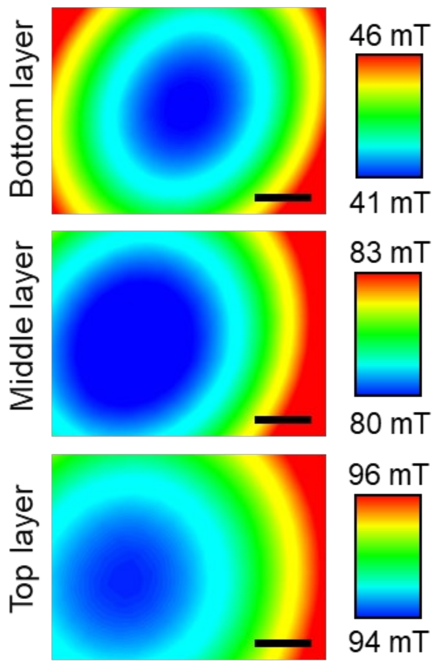

Figure S21. Simulated magnetic flux density of the P2, center of film with very small local magnetic field gap (1-3 mT). Scale bars represent 200 μm.

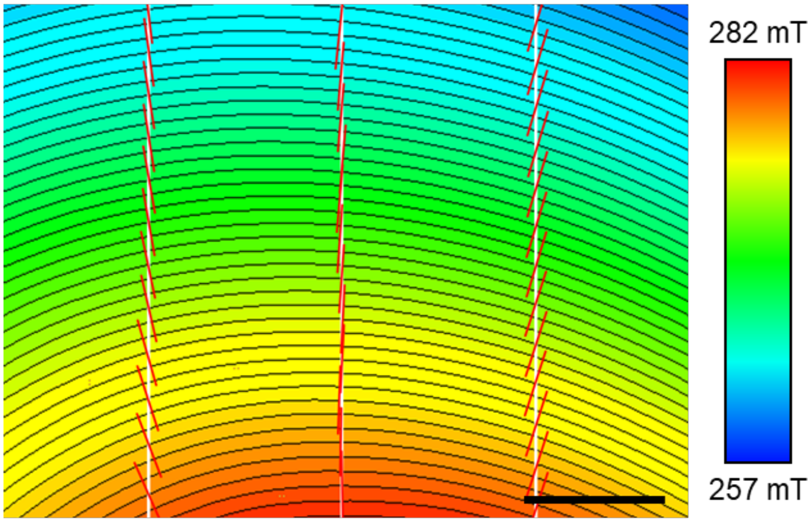

Figure S22. Simulated magnetic flux density of P1 position with three white vertical lines dividing four equally sized areas and red tangent lines. Black lines divide boundary area where have identical magnetic flux density. Scale bar: 200 μm.

More specifically, at the bottom layer of P1, the direction of magnetic flux density gradient directs to 6 o'clock direction. Then, at the middle layer of P1, the magnetic flux direction rotates 34° counterclockwise. Last, at the top layer of P1, the direction of magnetic flux density rotates 35° counterclockwise. Overall, the magnetic flux density gradient along bottom to top gradually rotated counterclockwise direction, 69°, thus creating a local vertical vortex in gap location.

The other outside of circular patterns, P1′ and P1″, where have right-handed chirality, also have counterclockwise, right-handed gradient changes as shown in P1′ and P1″ of **Figure 5b** and **5f**. However, the trends of gradient changes are different for different similar locations. The gradient changes from bottom to middle layer rotates further than middle to top layer. Specifically, the gradient at bottom layer of P1′ directs to 6 o'clock, then rotates 16° at middle layer and 2° at top layer toward counterclockwise, right-handed direction. In the case of P1″ area, the gradient of magnetic flux density at bottom layer directs to 10 o'clock direction. Then, at middle layer of P1″, the gradient of magnetic flux density rotates 13° and 3° at middle and top layer, respectively. These correlation between CD results and simulated magnetic flux density supports the counterclockwise, right-handed flow change forms the right-handed chirality of CNCs. Furthermore, the field gradient changes of P1′ and P1″ diminish significantly in comparison to P1 location that explains the decrease of g-factor as discussed above (**Figure 3e**).

On the one hand, the magnetic flux density gradient at P1‴ area, where has left-handed chirality, changes gradually to clockwise, left-handed direction as shown in **Figure 5b** and **5e**. The magnetic flux density gradient of bottom layer of P1‴ directs to 11.5 o'clock, then rotates 10° and 7° at middle and top layer, respectively, that may suggest the clockwise, left-handed flow vortex that results in traditional the left-handed chirality (**Figure 3e, 5b**). At P2, center of film, the direction change of magnetic field gradient has no trends due to radial pattern at the center and negligible gradient of 5 mT compared with other position (**Figure S21**).

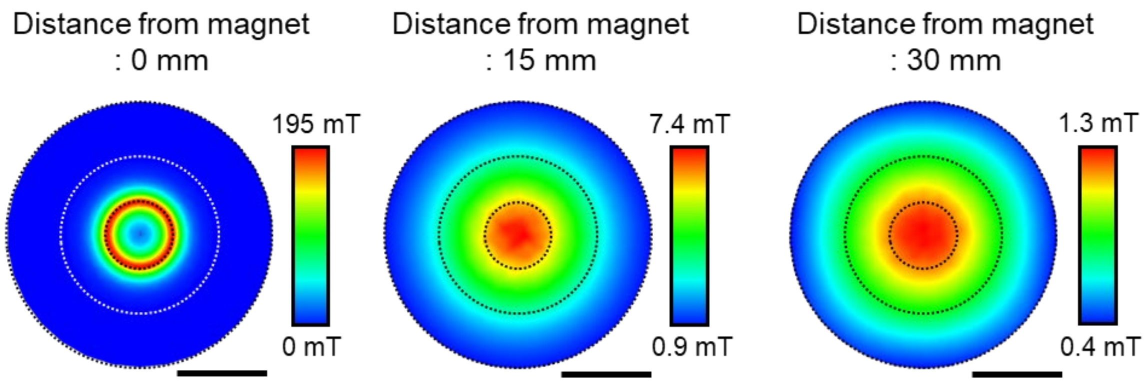

Figure S23. Simulated magnetic flux density of coin-shape magnet with different distance between petri dishes. Scale bars: 20 mm.

Table S1. Diffusion coefficients at different locations and layers (unit: cm^2/s)

D	Bottom	Middle	Top
P1	1.5×10^{-6}	2.3×10^{-6}	3.0×10^{-5}
P2	1.5×10^{-9}	8.2×10^{-8}	5.2×10^{-9}
P3	2.0×10^{-7}	-3.3×10^{-7}	-8.4×10^{-6}
No magnetic field	7.5×10^{-9}	-3.6×10^{-9}	3.1×10^{-8}

The diffusion coefficient at P1 location, is the highest with three orders of magnitude higher value than diffusion coefficient of freely dried CNC/MNP suspension without magnetic field (**Table S1**). This indicates that the flows at P1 provide sufficient shear to generate right-handed chirality. The diffusion coefficient at P2, center, was the lowest among P1-3 and similar with the diffusion coefficient of CNC/MNP suspension without magnet that indicates insufficient shear for the CNC alignment. The diffusion coefficients at P3, aggregated MNP area, are about one order magnitude lower than P1 and about one-two order magnitude higher than P2. However, regardless of D values, aggregated MNPs obstruct the assembly of CNCs during evaporation.
